# GARN: Sampling RNA 3D Structure Space with Game Theory and Knowledge-Based Scoring Strategies

**DOI:** 10.1371/journal.pone.0136444

**Published:** 2015-08-27

**Authors:** Mélanie Boudard, Julie Bernauer, Dominique Barth, Johanne Cohen, Alain Denise

**Affiliations:** 1 PRiSM, CNRS UMR 8144, Université de Versailles-St-Quentin-en-Yvelines, 78000 Versailles, France; 2 LRI, CNRS UMR 8623, Université Paris-Sud, 91405 Orsay, France; 3 AMIB, Inria Saclay-Ile de France, 91120 Palaiseau, France; 4 LIX, CNRS UMR 7161, Ecole Polytechnique, 91120 Palaiseau, France; 5 I2BC, CNRS, Université Paris-Sud, 91405 Orsay, France; University of Georgia, UNITED STATES

## Abstract

Cellular processes involve large numbers of RNA molecules. The functions of these RNA molecules and their binding to molecular machines are highly dependent on their 3D structures. One of the key challenges in RNA structure prediction and modeling is predicting the spatial arrangement of the various structural elements of RNA. As RNA folding is generally hierarchical, methods involving coarse-grained models hold great promise for this purpose. We present here a novel coarse-grained method for sampling, based on game theory and knowledge-based potentials. This strategy, GARN (Game Algorithm for RNa sampling), is often much faster than previously described techniques and generates large sets of solutions closely resembling the native structure. GARN is thus a suitable starting point for the molecular modeling of large RNAs, particularly those with experimental constraints. GARN is available from: http://garn.lri.fr/.

## Introduction

RNA molecules are involved in diverse biological processes in the cell. An understanding of the way in which RNA molecules adopt a 3D structure provides considerable insight in to the functional roles of these molecules. Methods for designing sequences so as to obtain specific functions are within reach [1]. An ability to design RNA molecules with a particular function is essential for therapeutics [2], but would also be very useful in emerging fields such as nanotechnology [3]. The structural building alphabet of RNA appears to be simpler than proteins alphabet, but the prediction of 3D structures for RNA has proved less straightforward than initially thought [4]. The structural diversity of RNA folds has made prediction a difficult task. However, the hierarchical nature of the RNA folding process [4–6] is the key to successful prediction strategies. Secondary structure prediction strategies [7–11] are very useful as a first step in modeling, because they often provide essential accurate information about the local base structure. Recently developed methods for the prediction of 3D structure for RNA [12–15] also make great use of the many studies on base interaction classification [16–21], by encoding base pairing and stacking both as energy functions [22–24] and in fragment libraries [17, 25].

These methods generate interesting samples for the analysis of experimental data [26, 27], but other techniques have shown that a broader sampling strategy can provide considerable insight into RNA function [28].

A few thousand RNA structures are now available from the Protein Data Bank (PDB), and these data have improved our understanding of RNA structures. They have increased the quality of energy potentials for RNA. This is true not only for the traditional force fields used in molecular dynamics simulation, but also for knowledge-based (KB) potentials. Initially developed for protein structure prediction [29–32], KB potentials have proven to be efficient for RNA structure prediction and sampling [30, 33–35].

The use of various levels of molecule representation is a major feature of most of these effective techniques [36]. Modeling can benefit from the inherent hierarchical nature of RNA, through the use of representations as coarse as secondary structure elements (SSEs). A graph-based representation can describe the structure of the molecule. Such representations of secondary structure are used in various settings [13, 28, 30, 37–41] and can account for 3D structure folding and dynamics.

We present here a new strategy for sampling RNA 3D structures, combining a coarse-grained graph-based representation, KB potentials, and game-theory algorithms. Each SSE is represented as one or a few nodes on a graph. The nodes are linked by covalent bond connections, and non-bonded interactions are represented with various types of KB potentials. Game theory is used to make the system evolve and to provide putative conformations: the nodes are the players of *sampling* games. With the exception of proof-of-concept studies for RNA backbones [38] and for various bioinformatics applications [42–44], game-theory approaches for RNA structure prediction have barely been studied. Game theory is a suitable tool for understanding systems in which the players have preferences for certain solutions. It favors local, egotistical choices rather than searching for a global optimum.

In this context, finding RNA conformations satisfying structural and potential constraints is seen as a local optimization problem in which each SSE or *player* (or set of players) tries to maximize its *payoff* function (which is equivalent to minimizing its energy function).

## 1 Materials & Methods

### 1.1 Overview

GARN combines a coarse-grained 3D representation, a knowledge-based (KB) scoring scheme, and efficient search techniques. The idea is that a stable solution, referred to as a Nash equilibrium in Game Theory, could be used to represent a stable 3D structure for RNA.

In game theory: (i) the strategy set of an action is called *the set of pure actions* available, (ii) an action can be a distribution law and (iii) a *mixed strategy* is an assignment of a probability to each pure action. This allows a player to select a pure action at random, according to the mixed strategy.

One of the key results of Game Theory is the Nash theorem [45]: every game with a finite number of players and a finite number of pure strategies has at most one Nash equilibrium in mixed strategies. A Nash equilibrium can be considered as a stable solution and can be interpreted as the state on which the system will converge if the players are rational. It is hard to find a Nash equilibrium [46], but methods have been designed to compute such equilibrium, and they can be used to understand the behavior of such systems and to make predictions.

One way to obtain a Nash equilibrium is to use simple algorithms in which each player selects a best response strategy, evaluated relative to the decisions taken by the other players. Probabilistic versions can be efficient, but major improvements have been achieved through studies of the dynamics of these algorithms [47].

Another approach is the *multi-armed bandit problem* [48], in which a player plays many times on different slot machines. At each step, the player chooses the machine to be played and receives a gain. The goal is to maximize the total gain, i.e., the sum of gains received at each pull, taking into account the history of previous pulls. When gains depend on a fixed probability law and pulls are independent, the results are measured in terms of expected loss. The multi-armed bandit problem can be resolved by a method of regret minimization [49], in which the expected loss after several rounds of play is minimized.

As with the multi-armed bandit problem, we use the regret minimization to solve our RNA folding problem. Basically, when searching for minima with game theory, the type of search performed is similar to that in force-field experiments. We used a reference set of RNA structures to construct a KB scoring framework for use in a lattice setting in which the players (RNA SSEs) evolve. We show that game theory strategies are efficient for sampling various conformations, particularly for large molecules with complex substructures, such as three-way junctions.

A schematic description of our approach is provided in S1 Fig. The players are built from the coordinates for coarse-grained players (i.e., SSEs) provided by the user. These coordinates can be obtained from modeling software or by prediction techniques [15, 17, 25, 33, 50], or even a crude model of the extended chain in which SSEs are represented. The whole molecule can thus be seen as a graph of players.

The 3D space is then modeled as a triangular lattice, as previously described [51]. Players are mapped onto the lattice and a scheme of evolution is applied. This scheme is derived from game theory and makes use of KB scoring. It provides a probabilistic encoding of the regions of the lattice on which the players are most likely to move at each step. The overall knowledge base is built from statistics for a *reference* dataset, with leave-one-out cross-validation, in which each PDB file of the *reference set* is assessed in turn, after its removal from the *reference set* to prevent bias in the statistical results.

Full atomic models are not reconstructed from GARN, and only coarse-grained models are compared. All-atom structures can be obtained and refined with other software, e.g., C2A [52], ModeRNA [15] or Assemble [50].

The sections below describe the methodology applied at each step and the evaluation scheme.

### 1.2 Data

Various 3D datasets can be used to establish statistical measurements for RNA structures [34, 35, 53]. We used a previously described stringent non-redundant dataset [34] that has been shown to be suitable for both all-atom and coarse-grained representation KB studies to perform the measurements, for both the setting up of players and lattice parameters. This dataset was also used for the KB scoring scheme.

This *reference set* contains 76 molecules for which secondary structures are available from RNA FRABASE [54] and the full 3D structure is available from the PDB.

For the evaluation of our approach and its comparison with other programs, we used two distinct datasets: the *evaluation set* and the *test set*. The *evaluation set* was obtained from the *reference set* by removing the structures that were too small, or that contained a four-way junction or a trailing unpaired chain that cannot yet be modeled. The *evaluation set* contained 38 structures. The structures of the *reference set* and the *evaluation set* are available from S1 Table. We also added 10 cases of structural and biological interest not present in the *reference set*. These cases formed the *test set*.

We assessed the performance of our method and compared it with the results of other studies on the *evaluation set* (S2 Table). We also performed a more detailed analysis on the *test set* (S3 Table).

### 1.3 Geometric representations

#### 1.3.1 Graph model

**Fig 1:**
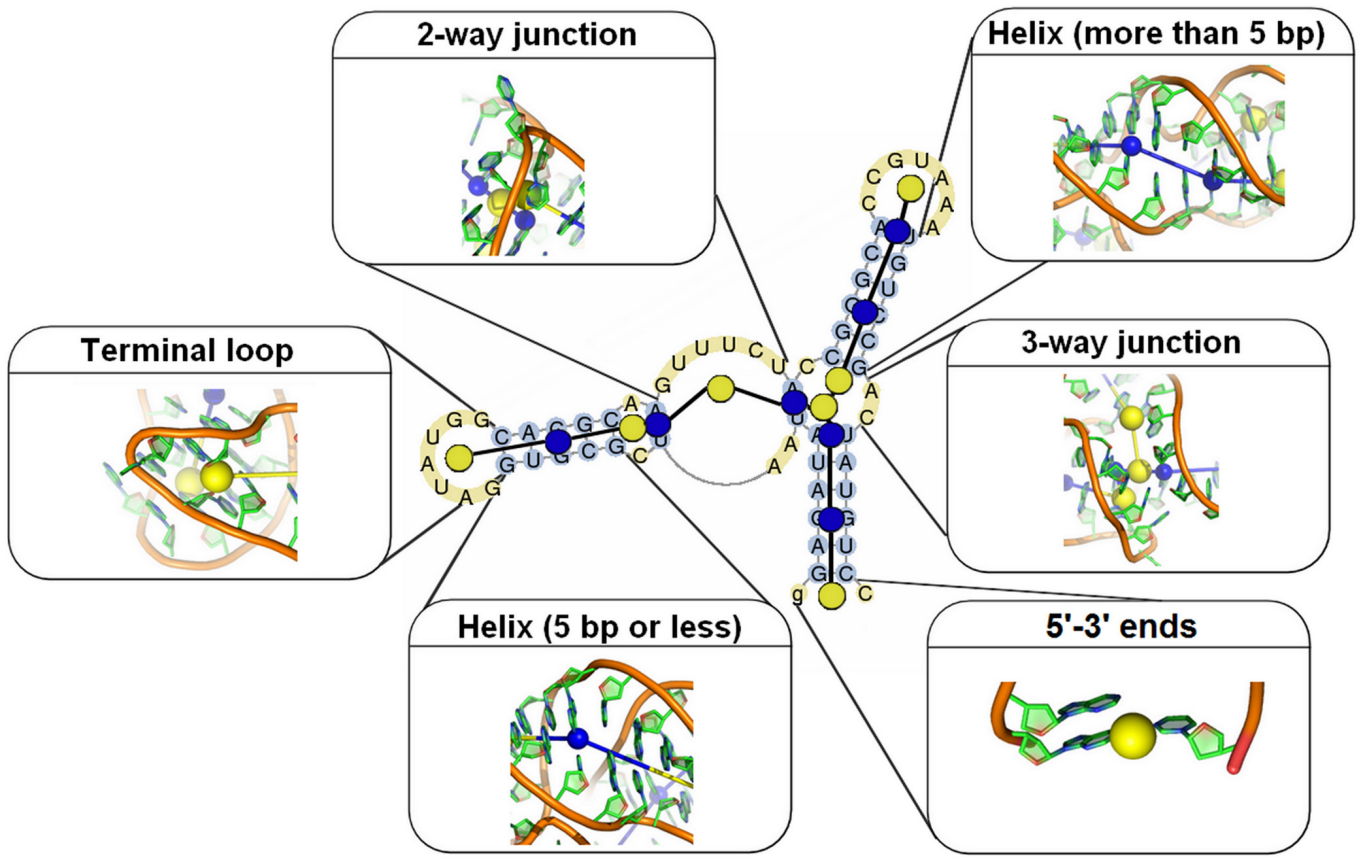
Graph representation of the xpt-pbuX guanine riboswitch aptamer domain (PDB ID: 4FE5). Nodes (players) are built from the secondary structure representation in which base-paired nucleotides are shown in blue and free nucleotides are shown in yellow. Blue nodes correspond to helices and yellow nodes to junctions. Each element, except the three-way junction is represented by one node (shown as a sphere), taken to be the geometric center of the heavy atoms. The three-way junction contains two nodes, accounting for helical stacking and branching, respectively.

The RNA molecule is represented by a graph similar to the representations previously used for other methods of RNA structure prediction [30, 37, 55]. The graph contains nodes representing SSEs, such as helices, junctions and loops, connected by edges representing the connectivity between these elements (See Fig 1). SSEs are represented by one or more nodes, also called *players*. The graph is built starting from the paired 5’-3’ ends and a depth first search is applied to find the SSEs and build the nodes.

- Each helix consisting of fewer than five base pairs is modeled using one player, taken to be the geometric center of all the heavy atoms considered. Longer helices are represented with as many players as required to keep a maximum of five base pairs per player and to account for possible long-range flexibility.
- For terminal loops (one-way junctions), bulges and two-way junctions [56], our representation uses one player, also located at the geometric center of the heavy atoms.
- Three-way junctions are modeled with two players. These players are defined such that one accounts for the helical stacking [57] and the other for the branching in the three-way junction (See Fig 1). The first player is located at the geometric center of the first base pair of the two stacked helices, and the second player is the geometric center of the heavy atoms in the junction.

Higher order junctions (e.g., four-way) are not natively included in this model, as large amounts of experimental 3D data were required to build and assess the model and the scores. In this study, we modeled one four-way junction with two three-way junctions and an additional *linker* player (i.e., a total of five players, see S2 Fig) as an example of the possibilities of extending this model to four-way junctions.

#### 1.3.2 Lattice

Each node (player) of the RNA model is set to lie on a 3D triangular regular lattice.

As explained by [51], the 3D triangular lattice provides a regular lattice (all lattice points have the same number of neighbors, and all pairs of adjacent lattice points are the same distance apart) and a high density (the 3D triangular lattice has a coordination number of 12, the cubic lattice has a coordination number of only 6). The triangular lattice is the best tradeoff between ease of counting for all the possible moves for each player and a flexible but folded structure.

We optimized the size of the lattice, by calculating all the distances between adjacent players in the graph for the *reference set*. A bimodal distribution was observed, with modes at 5.6 and 11.2 Å. For players at the same junction, the largest mode was 5.6 Å. The mode at 11.2 Å corresponds to adjacent players from different SSEs. A grid step size of 5.6 Å thus accommodates all cases.

Scoring parameters were also computed on the *reference set*. All distances between non-adjacent players were computed. All pairwise measurements were performed for the set of 239 players, and parameters were obtained from their distance distribution (see S3, S4 and S5 Figs). Scores were then normalized for experiments.

Unsurprisingly, the low-count regions (small distances) were difficult to deal with in the score evaluation. A Dirichlet Process Mixture was used to ease the process for Gaussian function evaluations [35].

### 1.4 Game

The game model contains: (i) players, corresponding to the nodes of the RNA graph, (ii) a set of possible strategies for each player, i.e., the spatial directions in which the next player can move, and (iii) player preferences, corresponding to the probability of each player choosing a strategy as a function of the previous moves of the other players, i.e., based on a score. From these settings, different game plays can be tested, to evaluate the best combinations allowing the system to evolve.

#### 1.4.1 Players and strategies

Each player has a set of 12 strategies, corresponding to the set of directions in the triangular lattice in which it is possible to move (See S6 Fig). The move gives the position of the next player, given that the distance between the player and the next player must remain constant. The set of strategies is thus different for different types of players:

- Players in small helices (fewer than 6 base pairs) can either stay in the same direction, or move at a maximum angle of 60° from that orientation.
- Players in large helices (more than 5 base pairs) and in small two-way junctions (one unpaired side being smaller than two nucleotides) can move in all 60° angle directions from their initial orientation. Large helices may also be *frozen*, i.e., allowed to move only by following the direction in which they are already oriented (See S7 Fig). This allows for some flexibility while restricting the conformational space.
- All possible moves in the lattice are allowed for one- and two-way junction players.
- While all possible moves in the lattice are possible for three-way junction players, once the first player, representing the stacking, has chosen a strategy (for the position of the second player), the second has to choose a position from the possible positions in the lattice.

The relative ordering of the players is determined as follows: each node of the graph is numbered according to a depth first search starting from the first junction (from the 5’-3’ paired ends) with the largest degree of branching (see S8 Fig). For three-way junctions, the unstacked helix is labeled first.

#### 1.4.2 Rules

As described above, the game uses a finite set of strategies for each player. It is also sequential, as players make their moves one after the other and the scheme is repeated several times, the previous configurations being known at each step (the game thus includes several turns for each player).

We use a regret minimization scheme: each player moves by choosing the most favorable and sufficiently likely environment, based on previous observations. We tried two different algorithms inspired by multi-armed bandits: the Upper Confidence Bound algorithm (UCB) [58] and the EXPonential EXPloration-EXPloitation algorithm (EXP3) [59].

In the UCB algorithm, each player chooses the strategy maximizing the sum of two scoring terms: (i) an *exploitation term*, corresponding to the mean of the previously obtained KB scores, including the current strategy and (ii) an *exploration term* favoring seldom-played favorable strategies. In the EXP3 algorithm, each player chooses a strategy from a Boltzmann distribution, the KB score being the Boltzmann energy of each state. The EXP3 algorithm is based on a Markov Chain Monte Carlo (MCMC) algorithm.

An action is allowed if it belongs to the set available to the specific type of player. An action is forbidden if: (i) two players occupy the same position on the lattice or (ii) two edges of the graph intersect. The set of possible actions available to a player is thus updated at each game turn, with the elimination of forbidden configurations.

#### 1.4.3 Gameplay

**Fig 2:**
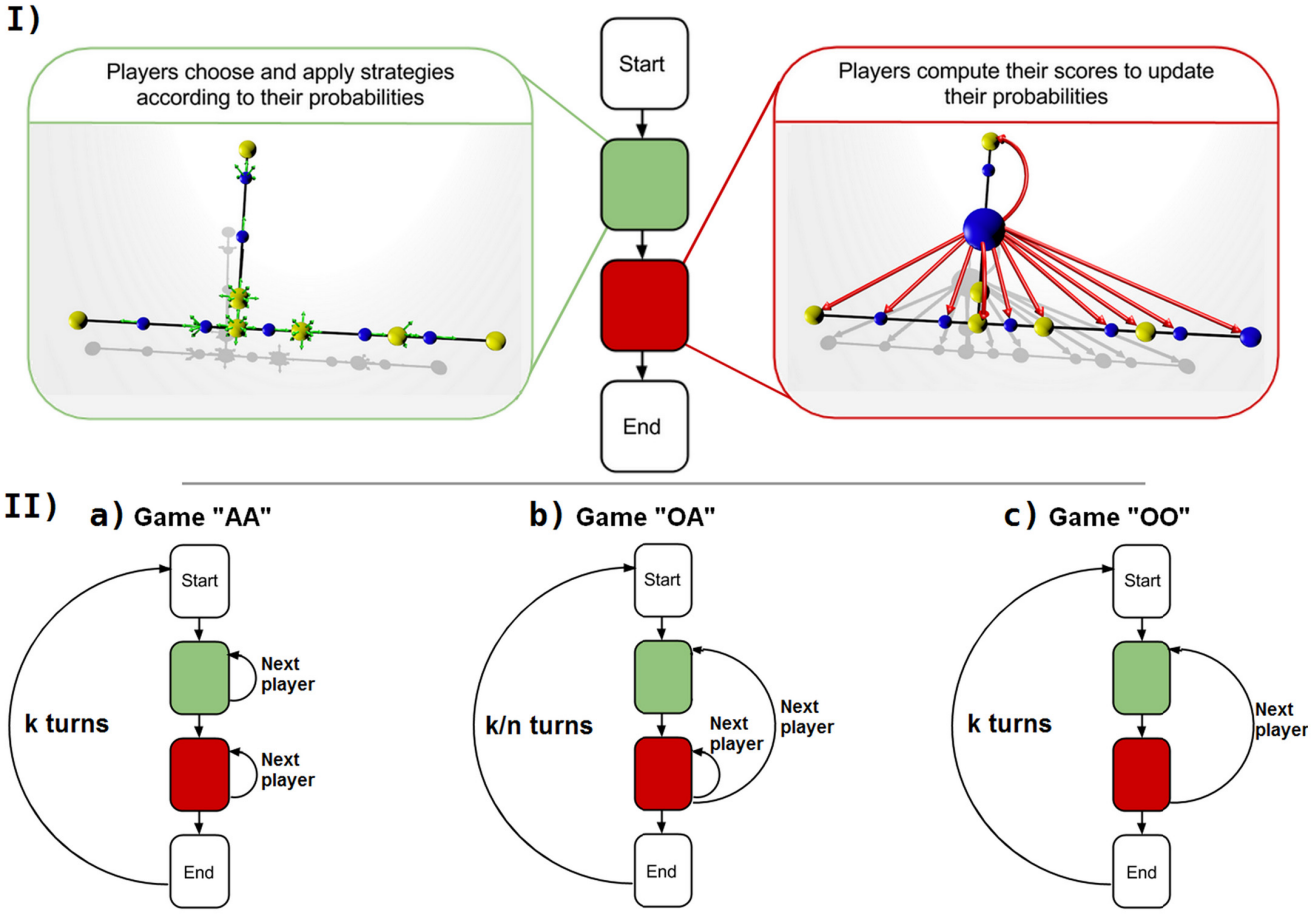
Overview of the gameplay. (I) Each game consists of several turns obeying a common set of rules. First, one or all the players choose a strategy (a direction on the grid) according to the relative probabilities of their choosing each strategy. The score is then updated by one or all the players, based on their distances from the other players, and the probabilities are then updated. Depending on the type of game, three schemes are possible (II): (a) in the AA game, all players apply a strategy and all players then calculate their scores, in two successive steps, (b) in the OA game, all players calculate their score each time a single player plays (the usual total number of turns *k* is thus divided by the number of players to allow for the same relative number of iterations), (c) in the OO game, each player applies a strategy and the score for that player is calculated before the next player plays.

Three different gameplays were tested, for sequential games, with each player acting in turn. These gameplays differ in terms of what the players do at each turn (see Fig 2): (AA) All players play, All players update; (OA) One player plays, All players update; (OO) One player plays, One player updates. In the OO game, playing corresponds to moving the next player on the grid and updating the probability for the following move (action).

### 1.5 Knowledge-based (KB) scoring

Once the game is set, with its players and strategies, a score is needed to define the welfare of a player in a specific conformation. Taking into account the coarse-grain nature of the model, we used KB-defined scoring functions to provide a pseudo-potential for SSEs. The scoring function is computed so as to mimic a KB energy: measurements of the distance *d* between two players are made and fitted to predefined functional forms. As SSE types do not have the same 3D characteristics, the scoring function parameters between two players depend on their respective SSE types. The *SSEtype*_*j*_ for player *j* may be a helix, a terminal loop, a two-way junction or a three-way junction.

Four different types of scoring functions were tested (see S5 Fig for an example):

- Lennard-Jones: $-A\cdot \left({\left(\frac{B}{d}\right)}^{12}-2\cdot {\left(\frac{B}{d}\right)}^{6}\right)$ where A and B depend on the player type (*SSEtype*). This score has only one mode.
- Modified Lennard-Jones: the positive repulsive part of the Lennard-Jones potential is flattened out to 0, so as to minimize local effects.
- Gauss: $A\cdot \frac{1}{\sigma \sqrt{2\pi }}e^{-\frac{{\left(d-\mu \right)}^{2}}{2\sigma^{2}}}$ where A, *σ* and *μ* depend on the player type (*SSEtype*). With a Gaussian mixture, the score has several modes.
- 1/*d* ^2^: a simple calculation of the inverse of the square distance.

Parameters for each scoring function were calculated with the *reference set* [34]. Different parameters were calculated for different types of SSE: a scoring function between a helix and a two-way junction does not have the same parameters as a scoring function between a helix and a terminal loop. For each pair of SSE types, the scoring function mode (depending on the parameters) represents different preferred distances between players. The scoring function is built as described by [35] and is taken to be the observed probability. To calculate the score of a given player *j*, we sum the scoring function value between this player and all other players.

$$Score_{j}=\sum_{i=1}^{p}ScoringFunction\left(SSEtype_{j},SSEtype_{i}\right)$$ (1)

where *p* is the number of players, and *j* is the player for which a score is calculated. This score is used to update the exploitation term of the algorithm.

### 1.6 Evaluation

#### 1.6.1 Assessment for each molecule

For each molecule, a graph representation must first be created. The secondary structure is obtained from RNA FRABASE [54]. For each 3D structure, the geometric center of each nucleotide is used to compute the coordinates of the nodes of the graph, as explained above. The Root Mean Square Deviation (RMSD) between structures is calculated from coarse-grained models containing only the nodes. The RMSD indicates the mean distance between the players of superimposed molecules. The RMSD is defined as:

$$RMSD\left(m,n\right)=\sqrt{\frac{1}{p}\overset{p}{\underset{i=1}{\sum }}||m_{i}-n_{i}|{|}^{2}}$$ (2)

where *m* and *n* are two molecules and *p* is the number of players, *m*_*i*_ the position of player *i* of the molecule *m* and *n*_*i*_ the position of player *i* of the molecule *n*.

#### 1.6.2 Evaluation of our game settings

Starting from a random conformation, different game settings were tested on the whole *evaluation set* and the *test set*.

The sampling results for the dataset are not given here, but S4, S5, S6, S7 and S8 Tables show the sampling results for some of the molecules of the *test set*. The conclusions below were drawn from the results for the *evaluation set*, highlighted for the *test set*.

#### 1.6.3 Comparison with other techniques

We evaluated the performance of this approach relative to other strategies, using the output PDB files, which we converted to our coarse-grained graph representation and used to calculate the RMSD. The inputs for these methods are the sequence and the same secondary structure used for GARN (when required by the method). We compared our results with those for four well known software suites: (i) iFOLDRNA [24](default server parameters), (ii) FARNA [17](from Rosetta3.2 w/ 50000 steps for small molecules (1MZP, 4TS0, 1E8O, 4FE5 and 4QJH) and 10000 steps for larger molecules (1LNG, 4WFL, 4QK8, 1MFQ and 4GXY) with the minimize RNA option) (iii) MCSym-MCFold [25](default server parameters), (iv) NAST [33](from circle conformation with 40000 steps and default parameters) and (v) RNAJAG [13](article data).

## 2 Results

### 2.1 Gameplays for generating insightful sample sets for analysis

#### 2.1.1 Choosing gameplay settings on the basis of molecule features

The previous exhaustive parameter and gameplay tests indicated that some settings were more appropriate for certain molecules, depending on their structural characteristics.

We extracted a rule of thumb for choosing the gameplay from the *evaluation set* according to the size of the molecule and the SSEs it contains. If the molecule contains no three-way junction, the settings are OA game/UCB algorithm/Modified Lennard-Jones potential/Helix frozen. If the molecule contains a three-way junction, the settings are AA game/EXP3 algorithm and the potential chosen depends on the helix/junction ratio. The potential is taken to be Lennard-Jones if the ratio is greater than 1.5, and Modified Lennard-Jones otherwise. S9 Table shows the correspondence between the ratio and the chosen gameplay.

**Fig 3:**
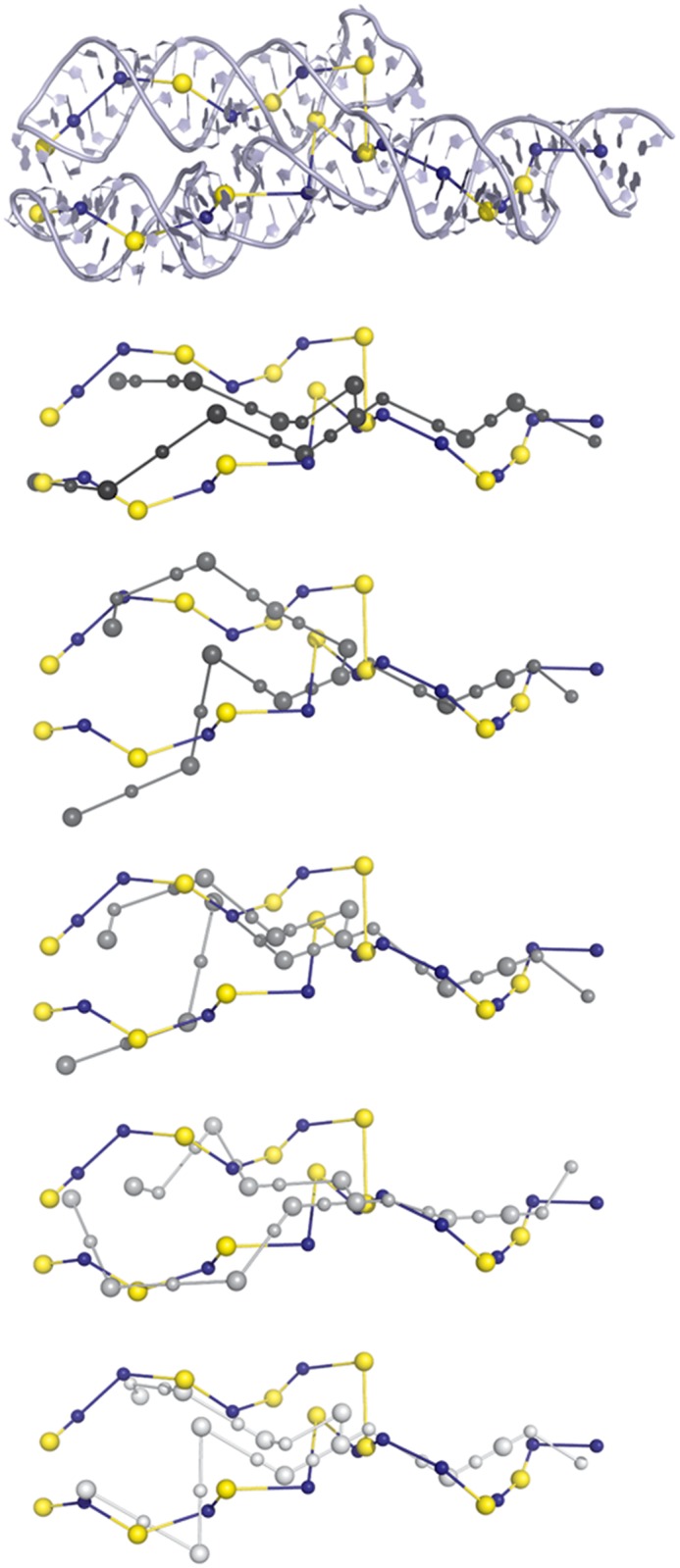
Visualization of five near-native samples for the 7S RNA of human SRP (PDB ID 1MFQ). The native structure graph is superimposed on the X-ray structure in the top panel. This superimposition indicates that the native structure graph (in blue and yellow) represents the native X-ray structure well. From top to bottom, the graphs most closely matching the native graphs are shown in gray (the darker the gray, the closer the match), superimposed on the native structure graph. These graphs show a good range of samples that could be used for reconstruction: the global shape of the molecule is recovered and the geometry of the junction is of interest.

**Fig 4:**
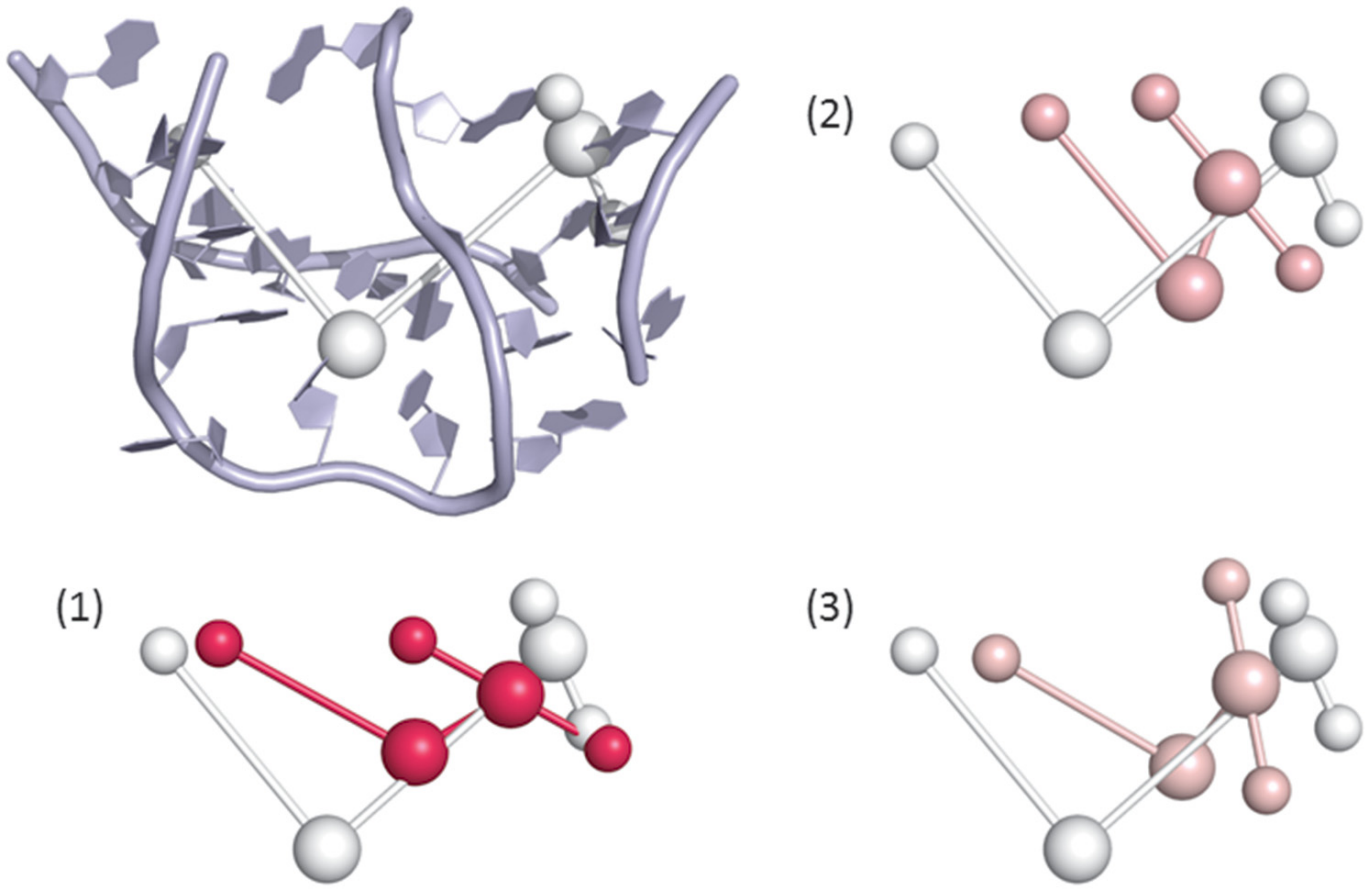
Three-way junction structure results for the 7S RNA of human SRP (PDB ID 1MFQ). The top left panel shows the structure and its associated GARN graph. Panel (1) shows the best three-way junction obtained with GARN (in pink) superimposed on the native structure graph (in white). Panels (2) and (3) show the second-best three-way junctions (in pink) superimposed on the native structure graph (in white).

We thus extracted three categories of molecules represented in the *test set*: (i) 1MZP and 4TS0 (ii) 1E8O, 4FE5, 1LNG and 4WFL and (iii) 4QJH, 4QK8, 1MFQ and 4GXY. S9 Fig shows sampling results for some of the molecules of the *test set*. For simple molecules, such as the 7SL RNA (PDB ID 1E8O), the global orientation of the junctions can be recovered with reasonable accuracy, despite the lack of tertiary contact information as an input. Fig 3 highlights some sampling results for the 7S RNA of human SRP. For more complex structures, such as the adenosylcobalamin riboswitch (PDB ID 4GXY), the best models obtained matched the known structures well. The complex geometry of the three-way junctions in the 7S RNA molecule was also well predicted, as shown in Fig 4.

#### 2.1.2 Influence of the settings

Each setting has an impact on the sampling. In the AA gameplay, each player waits for all the other players to change their positions before updating its probabilities. The game thus provides access to very different conformations between turns. Sampling is efficient as the difference between two consecutive steps is large. The OA gameplay also allows different conformations between consecutive steps, but as strategy is updated for only one player at a time, the sampling is less broad. In the OO gameplay, in which probabilities are updated for only one player, the other players have very little influence and sampling at each turn is purely local. Overall, these three gameplays cover different levels of aggressiveness for the sampling that can be fine-tuned for each molecule or experiment, depending on what needs to be achieved.

The performance of the algorithm used in the game depends principally on the type of scoring. The UCB algorithm worked better when the sampling and scoring did not involve a wide and rugged conformational space. The UCB algorithm is also known not to be very robust with noisy data [58]. By contrast, the EXP3 algorithm performed well for large molecules and a complex scoring scheme. This was expected for EXP3, as Gibbs/Boltzmann-based methods are known to be relatively robust to noisy data [59]. We also used the Linear Reward-Inaction algorithm (LRI, data not shown) [60], to determine whether pure Nash equilibria could be found, but the results were inconclusive.

The scoring schemes have various effects, mostly linked to the number of modes and the treatment of the repulsive part of the scheme. Both the Lennard Jones and Modified Lennard Jones schemes force players into a reasonable conformation, at least visually from a packing perspective, with only one mode in their definition distribution. However, the repulsive part of the Lennard Jones scoring function sometimes prevents the formation of tightly packed conformations of potentially interest from a biological perspective. A wider range of conformations is available with the Gauss score, which allows for different modes related to these conformations.

When a helix is frozen, i.e., when the helix cannot bend (See S7 Fig), its score is obtained by adding the score of its neighboring junctions divided by their distance on the grid and normalizing. This makes it possible to account for the influences of helices, which despite being rigid relative to their neighbors, lead to less compact structures. This is particularly relevant for smaller molecules, for which there is no need for compactness to be enforced.

We assessed the influence of the starting conformation, by using random initializations. We observed no marked impact of starting conformation on the conformations generated. In the AA game, after the first round, all players choose a conformation different from the starting conformation. In the OA and OO games, the starting conformation has an influence only on the first round, because all players choose a different conformation.

#### 2.1.3 Reaching an equilibrium

There is currently no way to demonstrate that a Nash equilibrium has been reached, but our modeling is based on potential games [61]: the maxima of the potential functions correspond to Nash equilibria. This situation is equivalent to the identification of a minimum energy corresponding to a stable structure in a physics-based energy function. However, the evolution of regret over time (or the number of turns/steps) suggests that the procedure can get close to the maximum, if such a maximum exists.

The regret is defined as:

$$Regret_{i}\left(T\right)=-\sum_{t=1}^{T}score_{i}\left(t\right)+\max(\sum_{i=1}^{T}score_{i}^{\prime }\left(t\right))$$ (3)

where *score*_*i*_(*t*) is the score of player *i* at time *t* and $score_{i}^{\prime }$ corresponds to the score with a fixed strategy *p*, with *p* ∈ 𝓐_*i*_, 𝓐_*i*_ being the set of strategies for player *i*.

The analogy with gradient descent is intuitively comprehensible, but it is hard to obtain formal proof [62–64]. The regret can take positive and negative values, but its amplitude should decrease over time to reach a stationary value corresponding to small steps around the maximum. S10 Fig shows the change in regret over time *t* (equivalent to the number of iterations) for several simulations. This figure shows that our simulations appear to reach a stationary amplitude, consistent with an equilibrium being reached.

### 2.2 The simple graph and game model allow for effective sampling

#### 2.2.1 A wide sampling to accommodate large molecules

For each molecule, we used the default gameplay of our procedure, as described above. Table 1 identifies the samples closest to native samples and shows the number of samples in a close range for a 50 samples generation run on these molecules. S10 Table provides the results for three different gameplays independently of the SSEs and junctions. Interestingly, classical approaches perform well for small molecules, but GARN performs at least as well as, or better than other strategies at the coarse-grained scale for larger molecules, as also shown in S2 Table.

**Table 1:** Comparison with other methods. Results from GARN runs with default parameters for full structures were compared with those for iFoldRNA, MCSym, FARNA and NAST. Not all servers can handle the ten structures of the *test set*, either due to the complexity of sampling or because the fragment templates are not available. For iFoldRNA, the only input provided to the server is the sequence. For NAST, only the secondary structure is used. The results obtained with NAST and iFoldRNA are therefore less accurate than those obtained with other techniques. NAST performs well on molecules of fewer than 50 nucleotides, but information about tertiary interactions is required to improve the results obtained. Our procedure not only yields better overall results for the minimal RMSD structure, it also provides several structures close to that of the native sample to choose from.

**PDB ID**	**# Nucleotides**	**# Players**	**RMSD**	**GARN**	**iFoldRNA**	**MCSym**	**Farna**	**NAST**
1MZP	55	8	min	4.32	8.23	NA	5.61	12.97
			max	10.58	12.68	NA	17.80	26.38
*# of samples with RMSD < 5Å (on 50 samples)*				*7*	*0*	*–*	*0*	*0*
1E8O	49	8	min	6.82	12.62	6.75	7.78	12.34
			max	15.40	17.71	15.55	21.02	20.29
*# of samples with RMSD < 8Å (on 50 samples)*				*13*	*0*	*1*	*1*	*0*
4FE5	67	14	min	7.14	15.04	NA	7.80	17.00
			max	14.53	20.12	NA	20.98	34.17
*# of samples with RMSD < 9Å (on 50 samples)*				*2*	*0*	*–*	*1*	*0*
4QJH	74	15	min	7.87	11.34	NA	8.99	23.44
			max	14.93	21.24	NA	18.67	26.57
*# of samples with RMSD < 9Å (on 50 samples)*				*3*	*0*	*–*	*1*	*0*
4TS0	89	21	min	10.42	NA	NA	9.47	19.84
			max	23.13	NA	NA	23.19	22.79
*# of samples with RMSD < 12Å (on 50 samples)*				*4*	*–*	*–*	*4*	*0*
1LNG	97	16	min	7.85	11.92	10.52	12.67	36.49
			max	17.07	35.53	29.58	30.19	59.66
*# of samples with RMSD < 10Å (on 50 samples)*				*6*	*0*	*–*	*0*	*0*
4WFL	107	18	min	8.82	18.08	NA	11.33	43.41
			max	16.22	25.75	NA	26.00	47.04
*# of samples with RMSD < 10Å (on 50 samples)*				*5*	*0*	*–*	*0*	*0*
4QK8	124	20	min	12.25	18.66	NA	13.23	54.43
			max	22.78	28.49	NA	22.19	59.44
*# of samples with RMSD < 14Å (on 50 samples)*				*3*	*0*	*–*	*1*	*0*
1MFQ	127	24	min	9.68	20.42	16.07	16.13	38.91
			max	20.64	34.08	30.97	41.27	44.17
*# of samples with RMSD < 11Å (on 50 samples)*				*7*	*0*	*–*	*0*	*0*
4GXY	172	32	min	14.27	NA	NA	15.84	69.04
			max	31.04	NA	NA	26.17	74.83
*# of samples with RMSD < 15Å (on 50 samples)*				*2*	*0*	*–*	*0*	*0*

Another advantage of GARN is that it does not require any fragment library to be available for SSEs of a specific sequence. Other strategies are also limited by the size of the molecules, due to computation costs. GARN can accommodate large structures, and the calculations for any given sample are very fast (30 min for a sample of 50 molecules for 4FE5, on a standard workstation or laptop). Comparisons of running times and of the number of conformations generated can be found in S11 Table.

**Fig 5:**
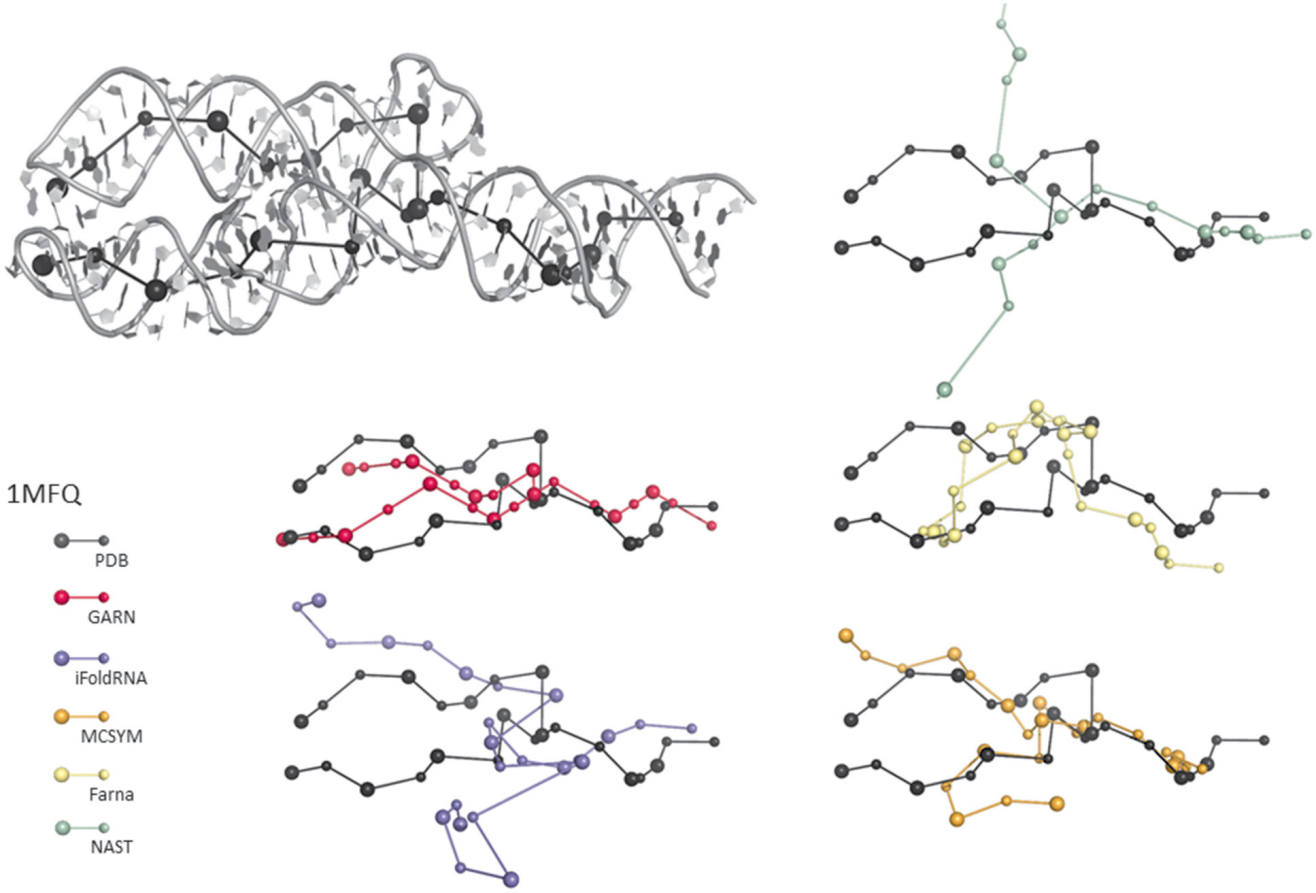
Comparison with other RNA structure prediction methods for the 7S RNA of human SRP (PDB ID 1MFQ). The best model generated by GARN (in pink) and the equivalent coarse-grained models obtained with other techniques are superimposed on the native structure graph (in black). The GARN technique does not enforce packing, but frequently provides the solution closest to the native structure.

Fig 5 shows that the structure predicted by GARN represents the coarse-grained biological structure of the molecule at least as well as the other techniques, for the 7S RNA of human SRP (PDB ID 1MFQ). Additional *test set* results can be found in S11 Fig. Overall packing is maintained and the level of structural information provided is as accurate as for the other methods. S2 Table shows a comparison for the whole *evaluation set*. Again, GARN performs at least as well as the other techniques.

The scoring used did not select the best candidate as the first solution, or even as one of the first five solutions, but the Energy vs. RMSD curve displays interesting behavior. S12 Fig shows the Energy vs. RMSD plots, using *Energy* = −*TotalScore* for all the methods using the default GARN scoring scheme. The *TotalScore* is the sum of the scores for all the players. S13 Fig shows the same plots, but with the default scoring system for each method. Both figures indicate that our scoring scheme does not provide an extremely sharp funnel towards low RMSD models, but that its scoring functions can be used in a coarse-grained setting. For example, our score discriminates well between badly packed structures from NAST or iFoldRNA and for large molecules (such as 4GXY). However, our score can not currently be used to sort our sampling.

#### 2.2.2 Highlight on three way junctions

Despite not having been specifically designed to handle three-way junctions and not including a detailed inventory of their conformations as input, our procedure proved useful for obtaining samples close to native samples. Fig 4 shows the results obtained for the 7S RNA of human SRP (PDB ID 1MFQ). Even when constrained by the lattice, the relative orientations of the junctions with the best scores were good. The structures of the three-way junctions showed that the directions obtained with GARN could be used in a hierarchical manner for the modeling of large molecules with coarse-grained representations.

Table 2 shows the RMSD results for three-way junction sampling for the test set obtained with four different techniques. GARN yielded good results for coarse-grained-only strategy, in some cases giving a RMSD value equivalent to that for fragment-based techniques (e.g., MCSym and FARNA). The minimal RMSD structure obtained with GARN was acceptable for reconstruction purposes, but was often further from the native structure than those obtained with MCSym and FARNA, which are known to be accurate high-resolution techniques. As our method is fast and not dependent on templates or consecutive SSEs, it provides structures that could later be refined.

**Table 2:** Comparison with other prediction methods for three-way junctions. The RNAJAG results are taken from [30] and were obtained with a different graph model. The RMSD values are, therefore, not directly comparable. Missing data indicate that the default server settings were unable to handle the request. The 4GXY four-way junction is represented by two three-way junctions. For each technique, 50 samples were requested.

**PDB ID**	**GARN**		**MCSym**		**Farna**		**NAST**		**iFoldRNA**		**RNAJAG**
	**Min**	**Max**	**Min**	**Max**	**Min**	**Max**	**Min**	**Max**	**Min**	**Max**	**Min**
1E8O	3.75	9.07	1.65	11.52	4.12	11.96	5.57	9.82	6.45	10.60	–
4FE5	2.79	4.77	–	–	1.23	5.87	1.95	12.35	4.80	11.12	–
4QJH	4.15	7.02	–	–	2.41	12.40	9.24	12.38	7.35	17.16	–
1LNG	7.19	7.19	3.76	6.85	3.02	10.31	6.05	36.56	3.79	9.67	9.04
4WFL 1	6.79	6.79	–	–	3.24	8.34	14.01	17.24	8.98	17.57	–
4WFL 2	5.65	8.83	–	–	4.64	12.25	11.26	14.40	7.46	14.11	–
4QK8 1	5.12	7.87	–	–	2.94	8.64	5.16	10.68	6.21	13.25	–
4QK8 2	3.95	8.29	–	–	3.82	10.13	8.62	12.20	5.35	14.75	–
1MFQ	4.70	5.71	2.87	6.06	5.31	7.5	9.26	19.71	18.16	27.13	5.26
4GXY 3-way	9.76	9.76	–	–	2.92	6.55	4.69	12.93	–	–	–
4GXY 4-way	9.57	13.31	–	–	6.50	11.87	16.55	39.84	–	–	–

We also compared our method with RNAJAG, for the nine molecules reported by [30]. S12 Table shows the results obtained. The RMSD values cannot be compared directly, as the two coarse-grained graph models are slightly different, but the results obtained were similar, suggesting that GARN can perform as well as RNAJAG.

## 3 Discussion

The sampling strategy implemented in GARN is based on the hypothesis that SSEs can be modeled spatially as players trying to maximize their own welfare. This hypothesis, although very coarse-grained, is consistent with hierarchical folding models based on advanced energy modeling techniques for RNA [40]. We thus used regret minimization algorithms, which can compute stable configurations. We also explored a range of knowledge-based coarse-grained models, to describe these configurations and their benefits. The experiments we performed showed that not all games and scoring models were equivalent for all molecules and that interesting traits can be highlighted with this approach. The two regret minimization algorithms for solving the multi-armed bandit problem implemented here, UCB and EXP3, differ in the efficiencies for finding solutions close to the native structure. This efficiency seemed to be correlated with the ratio of the number of helical SSEs and junction SSEs (i.e., players) and was not affected by terminal hairpins. The overall conformational space for the molecule is defined principally by the relative abundance of these SSEs and their arrangements, as highlighted by the relatively good results obtained for three-way junctions.

The choice of the scoring functions between SSEs appeared to be essential, particularly for the repulsive part of the scheme (close contacts). When a molecule contains a relatively large number of helical players, i.e., long helices, the repulsive part of the scheme is extremely important and close contacts must be avoided. Elongated conformations are preferred. However, the presence of a large number of junction elements calls for putatively packed structures best represented by a weak or non-existent repulsive part of the scoring system. This trade-off between packing and sampling must, artificially, be handled separately when dealing with simple sampling strategies for RNA molecules. It may also constitute a bias of the knowledge-based setup. This is a limitation of our model, in which decreasing the impact of the repulsive part of the model compensates for the rigidity imposed on the helical SSEs. However, it would not be expected to have a major effect on large-scale molecule sampling.

Three-way junctions slightly modify the configuration of the associated player, but they have a strong impact on the whole conformation. In our game model, this means that when one of these players changes its strategy, the score values may change for all the other players, from one turn to the next. The EXP3 algorithm was designed to be efficient in this type of environment and it performed better than the other algorithms tested. A Boltzmann-based strategy therefore appears to be appropriate for the modeling of more complex conformations, for which a suitable energy basin must be found in a potentially rugged landscape. By contrast, simple structures not containing three-way or higher-order junctions gave better results with the simple UCB algorithm, for which a slight change in the strategy of one player changed the scores of the other players. For simple structures, the various 3D conformations obtained were similar, and subtle changes could improve the better results. In this context, the UCB algorithm performs the equivalent of a local optimization in which the energy (scoring) space has to be smooth.

## 4 Conclusion and perspectives

The combination of methods inspired by game theory with knowledge-based models used in this study provided a good framework for sampling RNA molecules with a coarse-grained representation. The flexibility of the strategy described here resulted in a better performance than for existing techniques, for the sampling of medium-sized RNA molecules. The procedure is quick and easy and does not require large amounts of external information. Ideally, the different techniques could be combined for hierarchical or efficient local sampling, making it possible to build very large assemblies [28, 40]. Various external constraints could be added, such as experimental data obtained by electron microscopy or small-angle X-ray scattering, to obtain initial coarse-grained conformations for use as the input for high-resolution rebuilding and refinement, greatly accelerating the reconstruction process.

## Supporting Information

S1 Fig Overview. GARN has three parts: (i) the parameter setup for adjusting the game settings, (ii) the game process in GARN and (iii) comparisons with other published techniques.
(PDF)

S2 Fig Model for a four-way junction. Simple modeling of a four-way junction. Five players are used to model the four-way junction. They are located as if the junction consisted of two three-way junctions and a linker.
(PDF)

S3 Fig Score distances. Distances between all non-adjacent nodes for the *reference set*.
(PDF)

S4 Fig Lattice distances. Distances between adjacent nodes for all type of nodes, for the *reference set*.
(PDF)

S5 Fig Example of score. Distances between helix and two-way junction players in the *reference set*. **Red**: Raw distances. **Green** Lennard-Jones score. **Blue** Gauss score. The Gauss score tries to fit the KDE, and the Lennard-Jones score tries to identify a global best distance.
(PDF)

S6 Fig Strategies on the lattice. The current player, C, has to choose the direction of the next player, N, according to the direction of the previous player, P. If player C is frozen, this player can only choose the strategy corresponding to the black line; if not frozen, this player can choose to follow any of the blue lines.
(PDF)

S7 Fig Frozen and non-frozen helices. Mapping of frozen and non-frozen conformations: example of helix 0 and helix 1 of 1MFQ. The coarse-grained model (white) of the native structure is shown in the left panel. In the non-frozen mode (in pink), the lattice mapping is close to the native structure and allows for bending. In the frozen mode, in which the helices remain rigid (in blue), the lattice mapping forces the helix to remain straight.
(PDF)

S8 Fig Ordering of the players for the game. Players from the largest junction play first, and the other players are numbered according to a depth first search, starting from the first largest junction according to 5’-3’ ordering.
(PDF)

S9 Fig Visualization of near-native samples for the *test set*. The native structure graph is superimposed onto the X-ray structure. This superimposition shows that the native structure graph (in blue and yellow) represents the native X-ray structure well in each case. The graphs closest to those for the native structure are shown in gray (the darker the gray, the closer to the native structure), superimposed on the native structure. These graphs correspond to a good range of samples potentially useful for reconstruction: the global shape of the molecule is recovered and the junction has an interesting geometry.
(PDF)

S10 Fig Evolution of the regret. Evolution of the regret for a three-way junction player during the 4FE5 simulation. The top panel shows all strategies and the bottom panel shows four strategies. After 4000 steps, the amplitude of regret reaches a stationary value.
(PDF)

S11 Fig Comparison with other methods for predicting RNA structure. The best model generated by GARN (in pink) and the equivalent coarse-grained models obtained with other techniques (when available) are superimposed on the native structure graph (in black). The GARN technique does not enforce packing, but often provides the closest solution.
(PDF)

S12 Fig Energy vs. RMSD curves for the *test set*. Energy is calculated as the default GARN score multiplied by -1 and normalized.
(PDF)

S13 Fig Energy vs. RMSD curves for the *test set*. Energy vs. RMSD curves for the *test set*. Each plot shows the default energy scheme for each technique.
(PDF)

S1 Table Reference Set. The *reference set* contains 76 molecules of different sizes.
(PDF)

S2 Table Comparison of RMSD values. Comparison of the RMSD ranges obtained for different methods with the *evaluation set*. Minimum values of RMSD for each example are shown in blue.
(PDF)

S3 Table Test set. The *test set* contains 10 molecules of different sizes.
(PDF)

S4 Table Sampling results for the structure of the core of the ALU domain of the mammalian SRP (PDB ID 1E8O). The molecule contains 49 nucleotides and 8 players. Six different parameter sets per game type are shown. Values shown in blue highlight combinations providing conformations with RMSD values below 8Å. Elements highlighted in yellow correspond to the default GARN options for the molecule.
(PDF)

S5 Table Sampling results for the structure of the L1 protuberance in the ribosome (PDB ID 1MZP). The molecule contains 55 nucleotides and 8 players. Six different parameter sets per game type are shown. Values shown in blue highlight combinations providing conformations with RMSD values below 5Å. Elements highlighted in yellow correspond to the default GARN options for the molecule.
(PDF)

S6 Table Sampling results for the xpt-pbuX guanine riboswitch aptamer domain (PDB ID 4FE5). The molecule contains 67 nucleotides and 13 players. Six different parameter sets per game type are shown. Values shown in blue highlight combinations providing conformations with RMSD values below 8Å. Elements highlighted in yellow correspond to the default GARN options for the molecule.
(PDF)

S7 Table Sampling results for the structure of the core of the ternary S-domain complex of human signal recognition particle (PDB ID 1MFQ). The molecule contains 127 nucleotides and 24 players. Six different parameter sets per game type are shown. Values shown in blue highlight combinations providing conformations with RMSD values below 11Å. Elements highlighted in yellow correspond to the default GARN options for the molecule.
(PDF)

S8 Table Sampling results for the structure of the adenosylcobalamin riboswitch (PDB ID 4GXY). The molecule contains 172 nucleotides and 32 players. Four different parameter sets per game type are shown. As the molecule is relatively large, due to computing time constraints, extensive results are reported only for the AA game. Values shown in blue highlight combinations providing conformations with RMSD values below 15Å. Elements highlighted in yellow correspond to the default GARN options for the molecule.
(PDF)

S9 Table Gameplay settings for the test set. The default gameplay scheme is based on the existence of high-order junctions, and on the ratio of the number of players in helices to the number of players in junctions.
(PDF)

S10 Table Comparison with other methods. Elements in blue indicate the results for the GARN default settings.
(PDF)

S11 Table Computation time and number of output pdb files generated for all the compared methods. * indicates that the computation was performed locally on an Intel Xeon E5607 2.27GHz CPU. NAST computation was also performed with OpenCL on a NVIDIA Quadro 5000 GPU. Other computations were performed on dedicated servers. NAST appears to be much faster than the other methods, but it calculates only secondary structure interactions: the lack on tertiary information as input only allows for extended structures as a result. The computation time for RNAJAG was not available from [30].
(PDF)

S12 Table Comparison with RNAJAG for three-way junctions. GARN RMSD values are calculated only for the coarse-grained representation of the three-way junction. RNAJAG RMSD values were obtained from [30]. Elements in blue had the lowest RMSD values.
(PDF)
