# Supplementary material for: GARN: Sampling RNA 3D Structure Space with Game Theory and Knowledge-Based Scoring Strategies
Source: PLoS One. 2015 Aug 27;10(8):e0136444. doi: 10.1371/journal.pone.0136444 (PMC4551674; doi:10.1371/journal.pone.0136444)
Supplement: S1 Fig — GARN has three parts: (i) the parameter setup for adjusting the game settings, (ii) the game process in GARN and (iii) comparisons with other published techniques. (PDF) [file pone.0136444.s001.pdf]

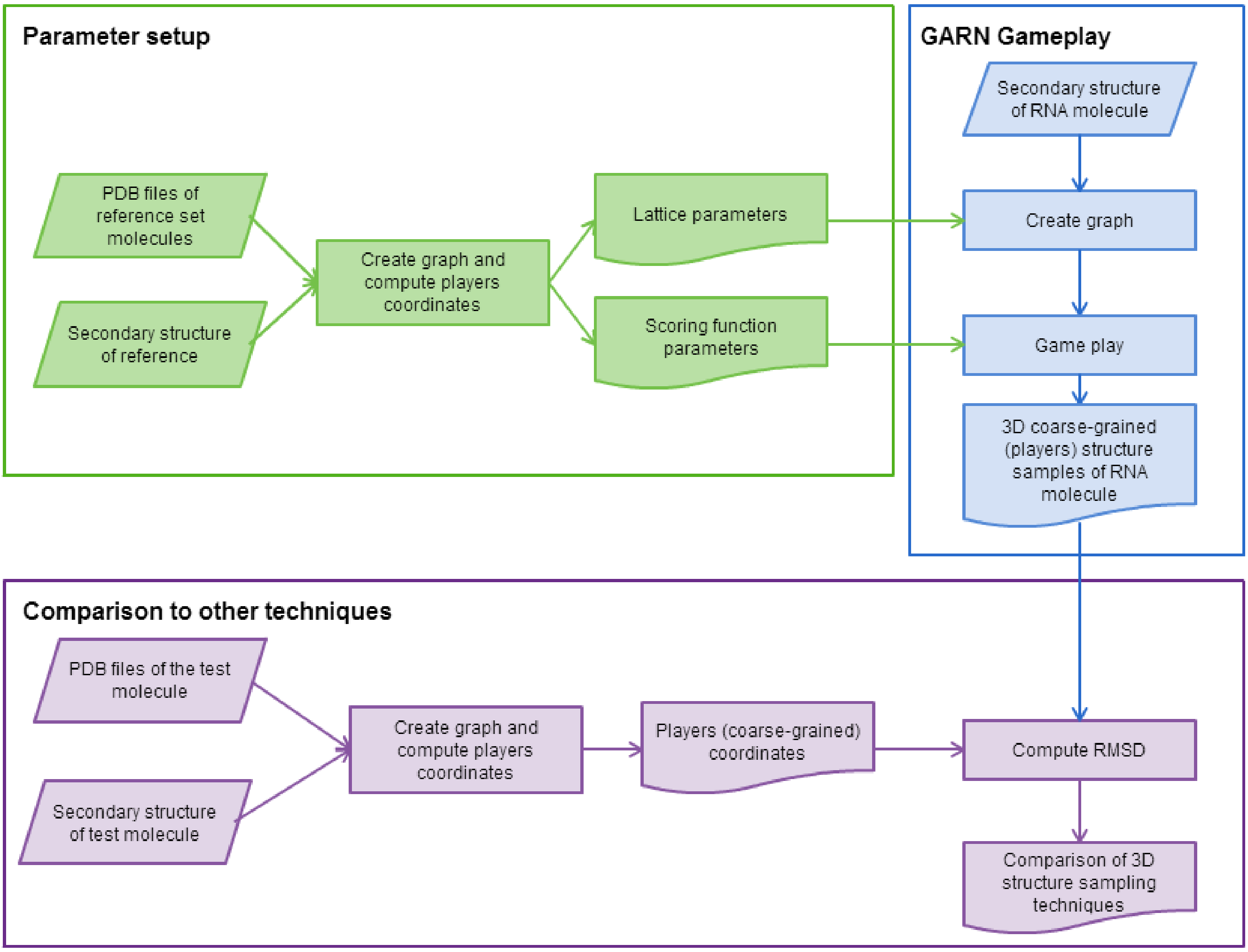

Figure S1: **Overview.** GARN has three parts: (i) the parameter setup for adjusting the game settings, (ii) the game process in GARN and (iii) comparisons with other published techniques.
