# Supplementary material for: GARN: Sampling RNA 3D Structure Space with Game Theory and Knowledge-Based Scoring Strategies
Source: PLoS One. 2015 Aug 27;10(8):e0136444. doi: 10.1371/journal.pone.0136444 (PMC4551674; doi:10.1371/journal.pone.0136444)
Supplement: S2 Fig — Simple modeling of a four-way junction. Five players are used to model the four-way junction. They are located as if the junction consisted of two three-way junctions and a linker. (PDF) [file pone.0136444.s002.pdf]

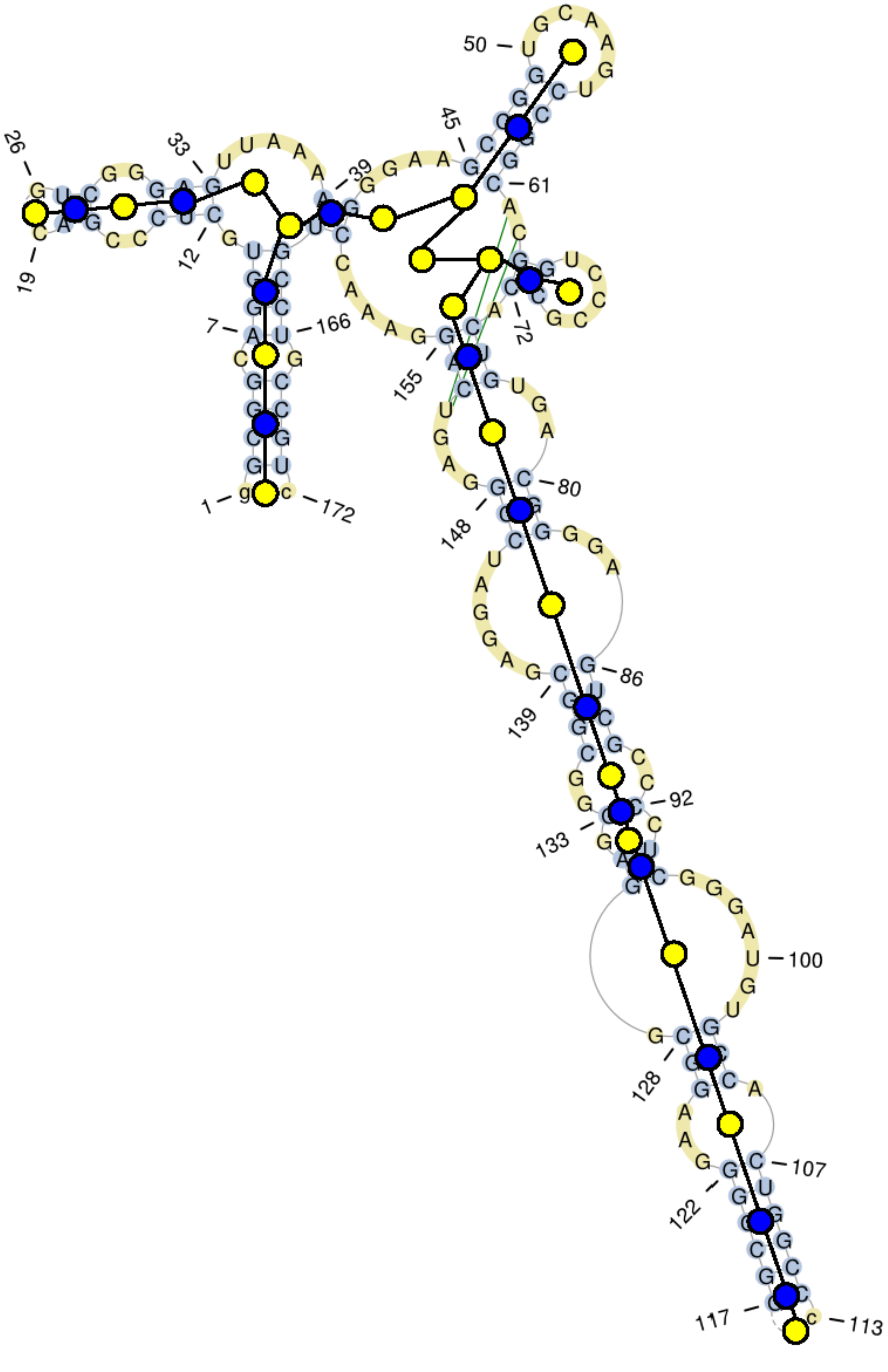

Figure S2: **Model for a four-way junction.** Simple modeling of a four-way junction. Five players are used to model the four-way junction. They are located as if the junction consisted of two three-way junctions and a linker.
