# Supplementary figures and images for: GARN: Sampling RNA 3D Structure Space with Game Theory and Knowledge-Based Scoring Strategies
Source: PLoS One. 2015 Aug 27;10(8):e0136444. doi: 10.1371/journal.pone.0136444 (PMC4551674; doi:10.1371/journal.pone.0136444)

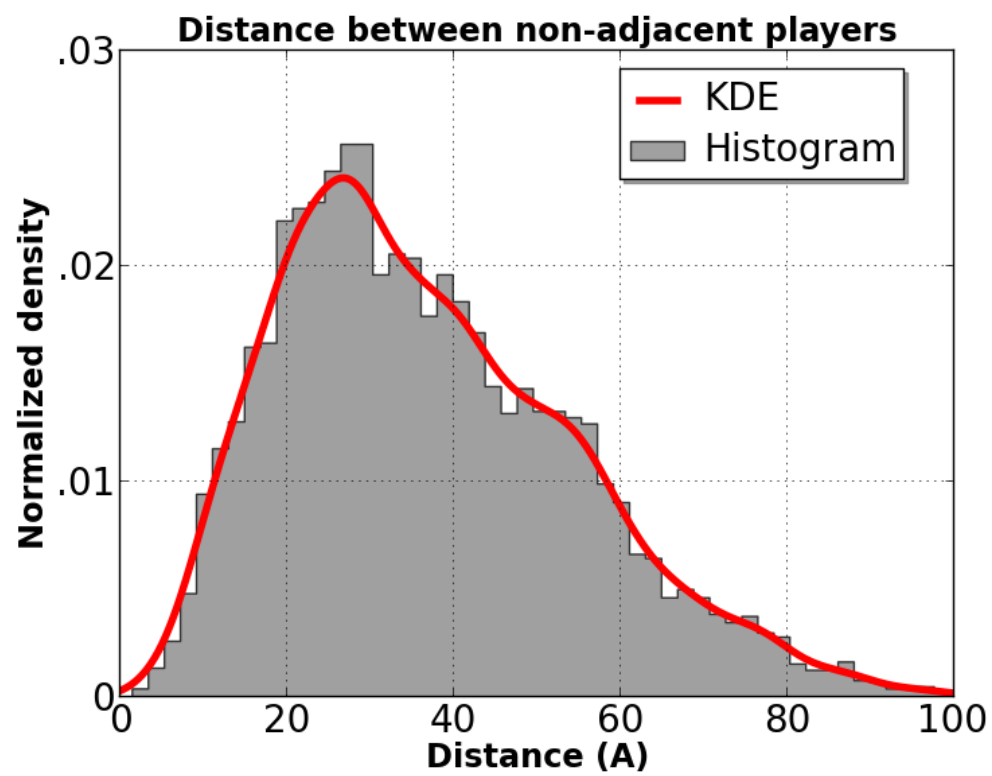

Figure S3: **Score distances.** Distances between all non-adjacent nodes for the *reference set*.

Supplement: S3 Fig — Distances between all non-adjacent nodes for the reference set. (PDF) [file pone.0136444.s003.pdf]
