# Supplementary material for: GARN: Sampling RNA 3D Structure Space with Game Theory and Knowledge-Based Scoring Strategies
Source: PLoS One. 2015 Aug 27;10(8):e0136444. doi: 10.1371/journal.pone.0136444 (PMC4551674; doi:10.1371/journal.pone.0136444)
Supplement: S4 Fig — Distances between adjacent nodes for all type of nodes, for the reference set. (PDF) [file pone.0136444.s004.pdf]

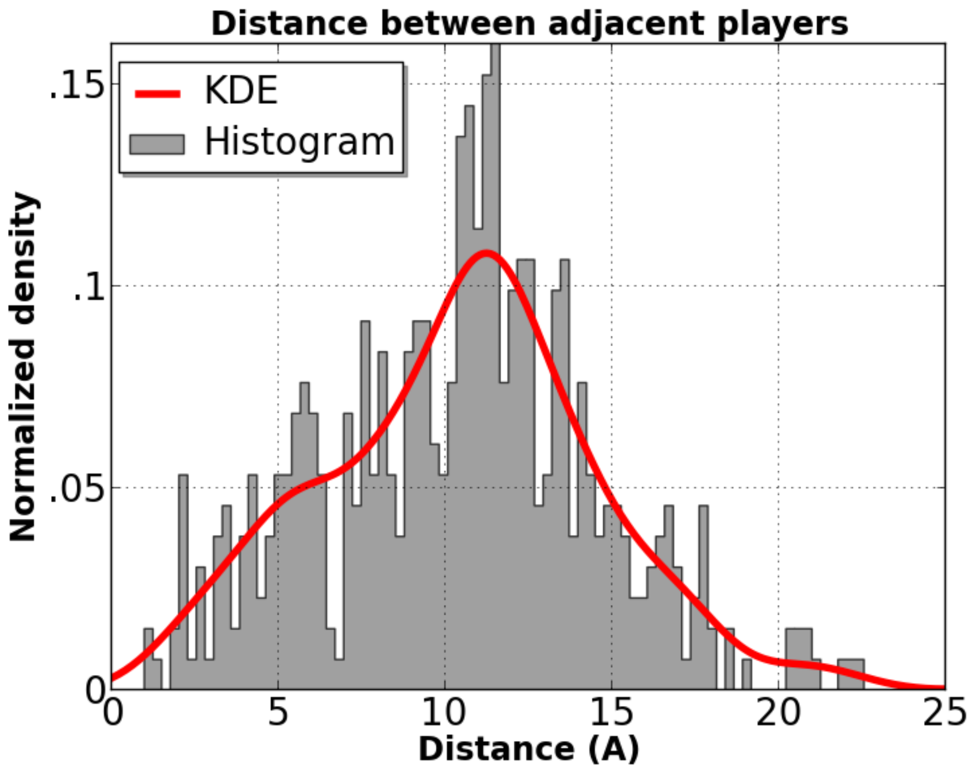

Figure S4: **Lattice distances.** Distances between adjacent nodes for all type of nodes, for the *reference set*.
