# Supplementary material for: GARN: Sampling RNA 3D Structure Space with Game Theory and Knowledge-Based Scoring Strategies
Source: PLoS One. 2015 Aug 27;10(8):e0136444. doi: 10.1371/journal.pone.0136444 (PMC4551674; doi:10.1371/journal.pone.0136444)
Supplement: S5 Fig — Distances between helix and two-way junction players in the reference set. Red: Raw distances. Green Lennard-Jones score. Blue Gauss score. The Gauss score tries to fit the KDE, and the Lennard-Jones score tries to identify a global best distance. (PDF) [file pone.0136444.s005.pdf]

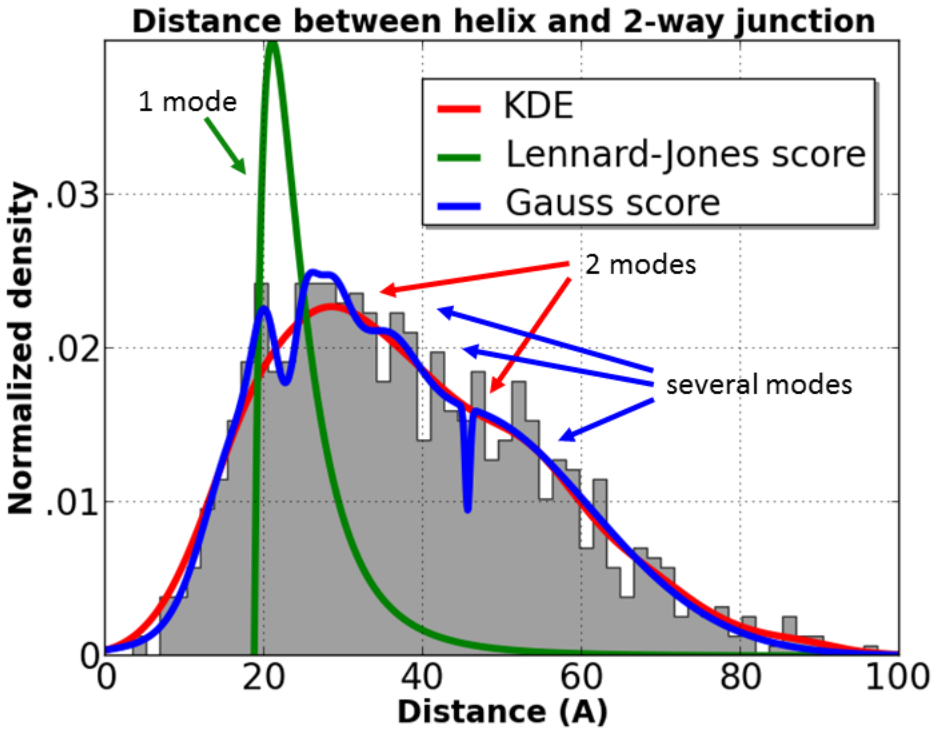

Figure S5: **Example of score.** Distances between helix and two-way junction players in the *reference set*. **Red**: Raw distances. **Green** Lennard-Jones score. **Blue** Gauss score. The Gauss score tries to fit the KDE, and the Lennard-Jones score tries to identify a global best distance.
