# Supplementary material for: GARN: Sampling RNA 3D Structure Space with Game Theory and Knowledge-Based Scoring Strategies
Source: PLoS One. 2015 Aug 27;10(8):e0136444. doi: 10.1371/journal.pone.0136444 (PMC4551674; doi:10.1371/journal.pone.0136444)
Supplement: S6 Fig — The current player, C, has to choose the direction of the next player, N, according to the direction of the previous player, P. If player C is frozen, this player can only choose the strategy corresponding to the black line; if not frozen, this player can choose to follow any of the blue lines. (PDF) [file pone.0136444.s006.pdf]

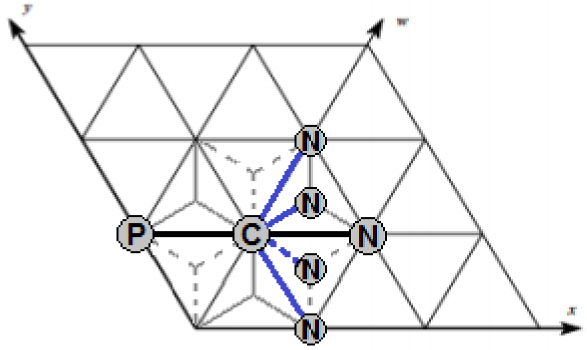

Figure S6: **Strategies on the lattice.** The current player, C, has to choose the direction of the next player, N, according to the direction of the previous player, P. If player C is frozen, this player can only choose the strategy corresponding to the black line; if not frozen, this player can choose to follow any of the blue lines.
