# Supplementary material for: GARN: Sampling RNA 3D Structure Space with Game Theory and Knowledge-Based Scoring Strategies
Source: PLoS One. 2015 Aug 27;10(8):e0136444. doi: 10.1371/journal.pone.0136444 (PMC4551674; doi:10.1371/journal.pone.0136444)
Supplement: S7 Fig — Mapping of frozen and non-frozen conformations: example of helix 0 and helix 1 of 1MFQ. The coarse-grained model (white) of the native structure is shown in the left panel. In the non-frozen mode (in pink), the lattice mapping is close to the native structure and allows for bending. In the frozen mode, in which the helices remain rigid (in blue), the lattice mapping forces the helix to remain straight. (PDF) [file pone.0136444.s007.pdf]

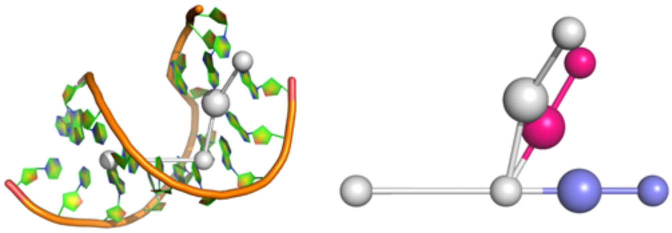

Figure S7: **Frozen and non-frozen helices.** Mapping of frozen and non-frozen conformations: example of helix 0 and helix 1 of 1MFQ. The coarse-grained model (white) of the native structure is shown in the left panel. In the non-frozen mode (in pink), the lattice mapping is close to the native structure and allows for bending. In the frozen mode, in which the helices remain rigid (in blue), the lattice mapping forces the helix to remain straight.
