# Supplementary material for: GARN: Sampling RNA 3D Structure Space with Game Theory and Knowledge-Based Scoring Strategies
Source: PLoS One. 2015 Aug 27;10(8):e0136444. doi: 10.1371/journal.pone.0136444 (PMC4551674; doi:10.1371/journal.pone.0136444)
Supplement: S8 Fig — Players from the largest junction play first, and the other players are numbered according to a depth first search, starting from the first largest junction according to 5’-3’ ordering. (PDF) [file pone.0136444.s008.pdf]

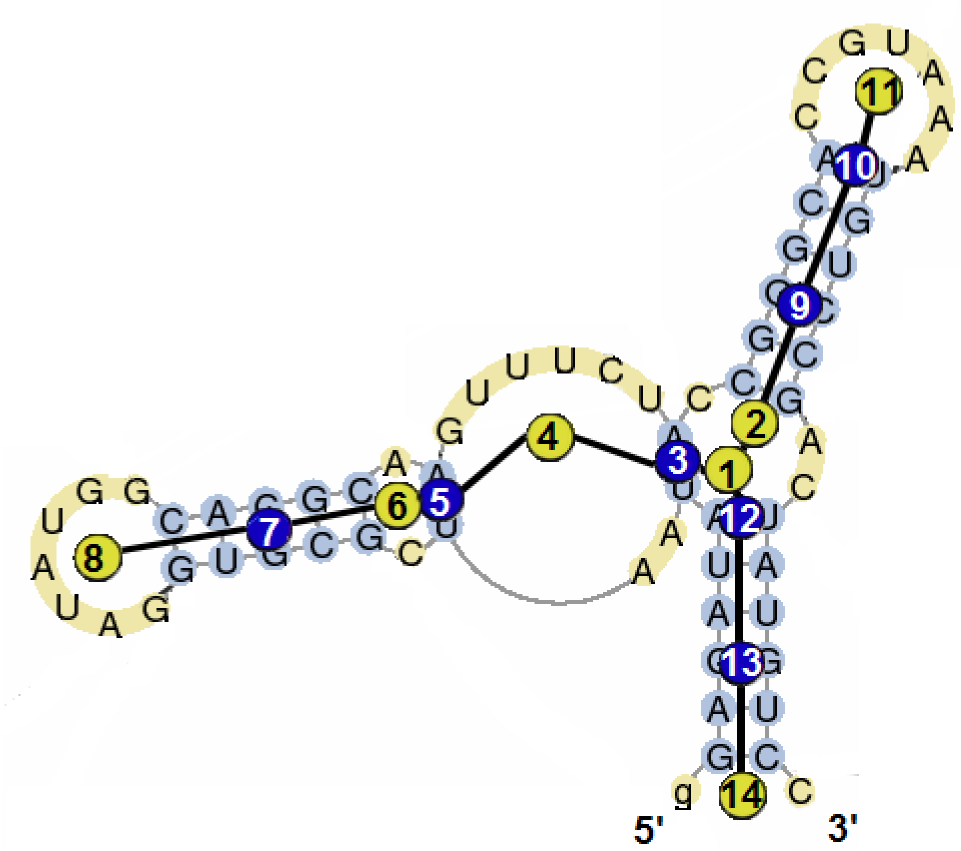

Figure S8: **Ordering of the players for the game.** Players from the largest junction play first, and the other players are numbered according to a depth first search, starting from the first largest junction according to 5'-3' ordering.
