# Supplementary material for: GARN: Sampling RNA 3D Structure Space with Game Theory and Knowledge-Based Scoring Strategies
Source: PLoS One. 2015 Aug 27;10(8):e0136444. doi: 10.1371/journal.pone.0136444 (PMC4551674; doi:10.1371/journal.pone.0136444)
Supplement: S9 Fig — The native structure graph is superimposed onto the X-ray structure. This superimposition shows that the native structure graph (in blue and yellow) represents the native X-ray structure well in each case. The graphs closest to those for the native structure are shown in gray (the darker the gray, the closer to the native structure), superimposed on the native structure. These graphs correspond to a good range of samples potentially useful for reconstruction: the global shape of the molecule is recovered and the junction has an interesting geometry. (PDF) [file pone.0136444.s009.pdf]

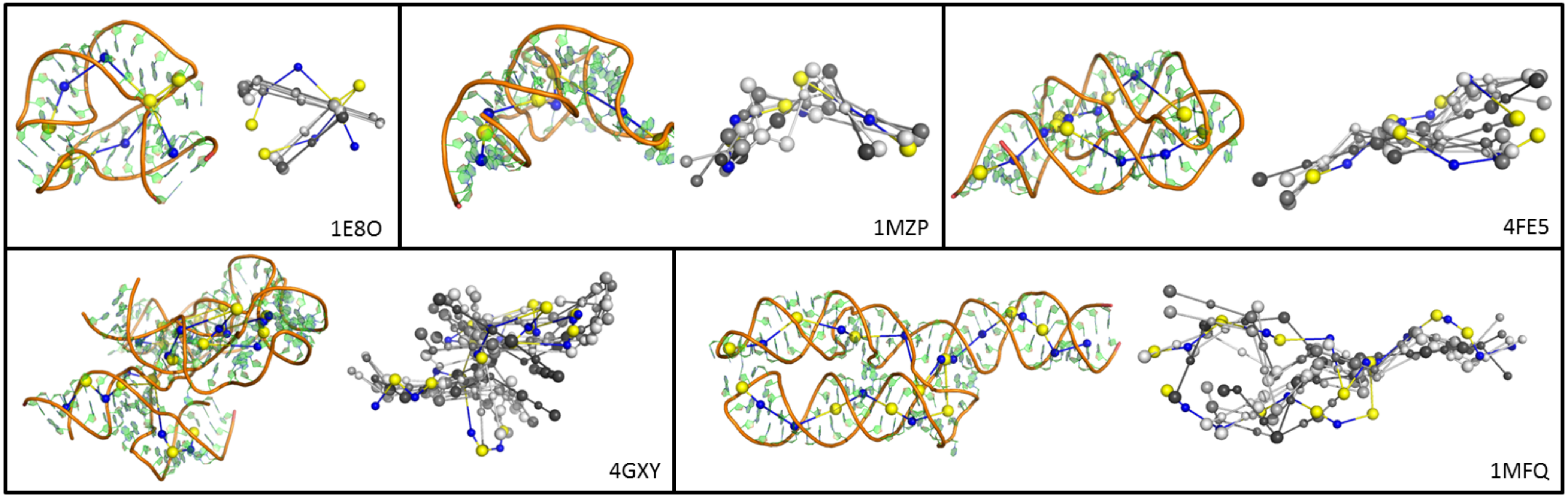

Figure S9: **Visualization of near-native samples for the *test set*.** The native structure graph is superimposed onto the X-ray structure. This superimposition shows that the native structure graph (in blue and yellow) represents the native X-ray structure well in each case. The graphs closest to those for the native structure are shown in gray (the darker the gray, the closer to the native structure), superimposed on the native structure. These graphs correspond to a good range of samples potentially useful for reconstruction: the global shape of the molecule is recovered and the junction has an interesting geometry.
