# Supplementary material for: GARN: Sampling RNA 3D Structure Space with Game Theory and Knowledge-Based Scoring Strategies
Source: PLoS One. 2015 Aug 27;10(8):e0136444. doi: 10.1371/journal.pone.0136444 (PMC4551674; doi:10.1371/journal.pone.0136444)
Supplement: S10 Fig — Evolution of the regret for a three-way junction player during the 4FE5 simulation. The top panel shows all strategies and the bottom panel shows four strategies. After 4000 steps, the amplitude of regret reaches a stationary value. (PDF) [file pone.0136444.s010.pdf]

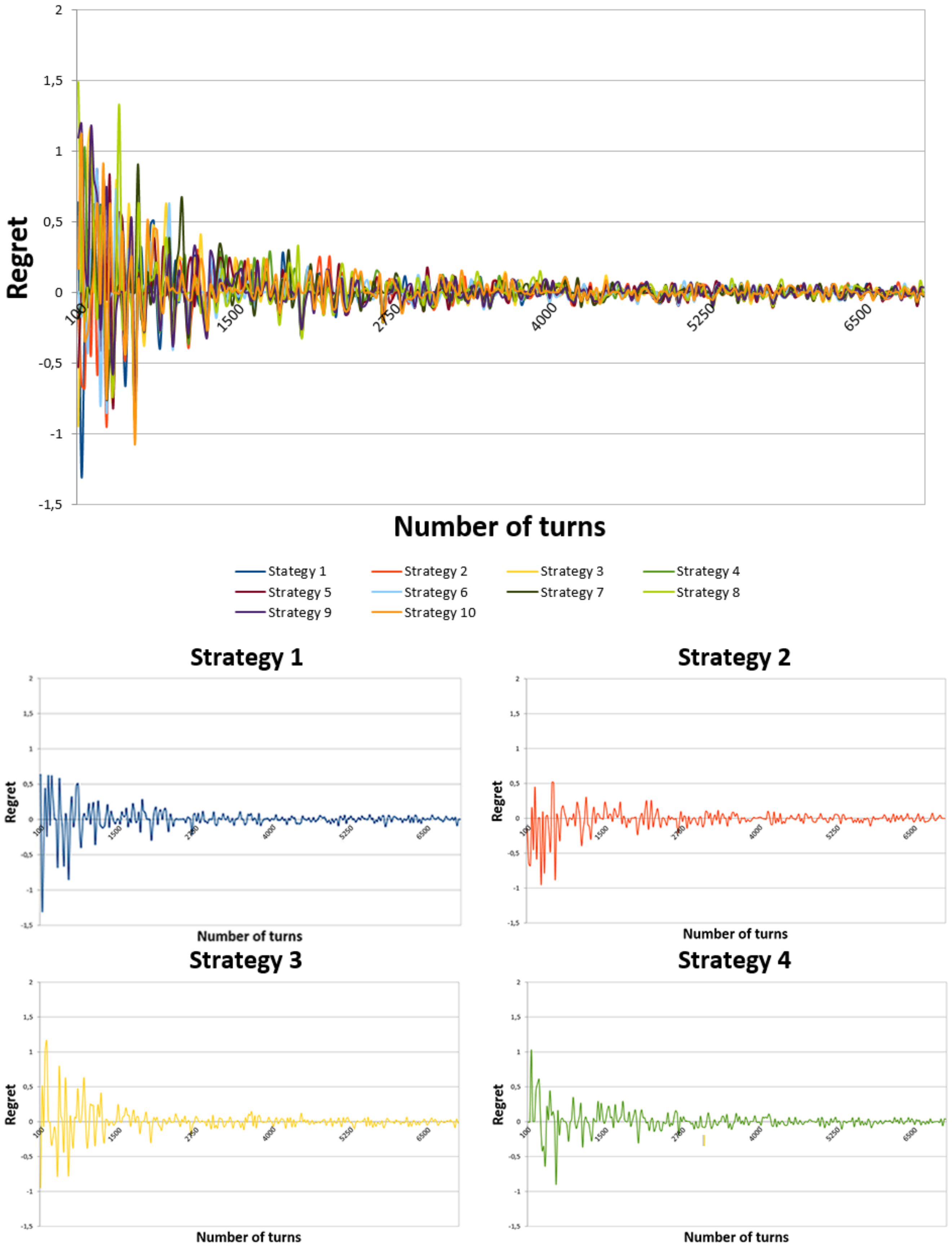

Figure S10: **Evolution of the regret.** Evolution of the regret for a three-way junction player during the 4FE5 simulation. The top panel shows all strategies and the bottom panel shows four strategies. After 4000 steps, the amplitude of regret reaches a stationary value.
