# Supplementary material for: GARN: Sampling RNA 3D Structure Space with Game Theory and Knowledge-Based Scoring Strategies
Source: PLoS One. 2015 Aug 27;10(8):e0136444. doi: 10.1371/journal.pone.0136444 (PMC4551674; doi:10.1371/journal.pone.0136444)
Supplement: S11 Fig — The best model generated by GARN (in pink) and the equivalent coarse-grained models obtained with other techniques (when available) are superimposed on the native structure graph (in black). The GARN technique does not enforce packing, but often provides the closest solution. (PDF) [file pone.0136444.s011.pdf]

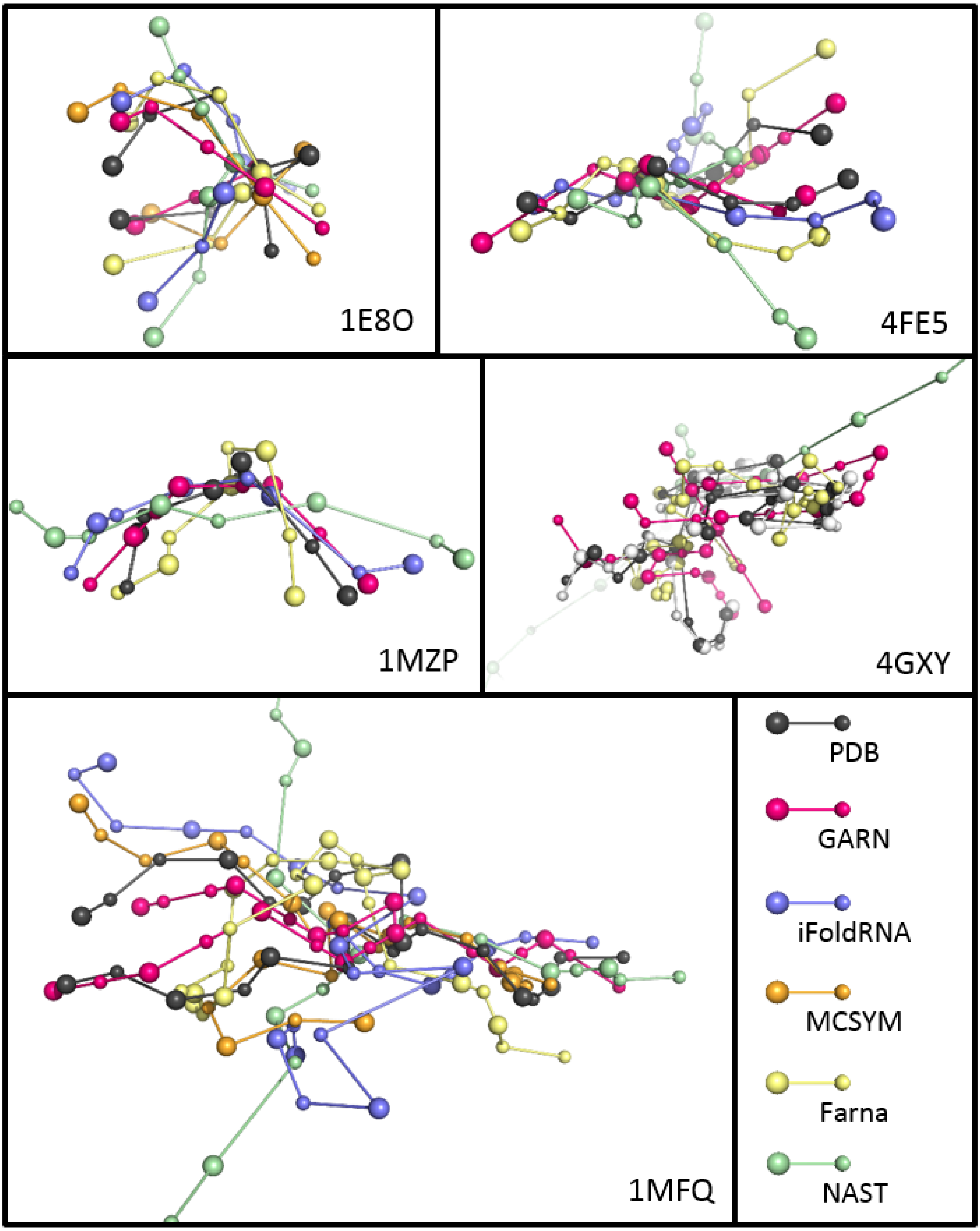

Figure S11: **Comparison with other methods for predicting RNA structure.** The best model generated by GARN (in pink) and the equivalent coarse-grained models obtained with other techniques (when available) are superimposed on the native structure graph (in black). The GARN technique does not enforce packing, but often provides the closest solution.
