# Supplementary material for: GARN: Sampling RNA 3D Structure Space with Game Theory and Knowledge-Based Scoring Strategies
Source: PLoS One. 2015 Aug 27;10(8):e0136444. doi: 10.1371/journal.pone.0136444 (PMC4551674; doi:10.1371/journal.pone.0136444)
Supplement: S12 Fig — Energy is calculated as the default GARN score multiplied by -1 and normalized. (PDF) [file pone.0136444.s012.pdf]

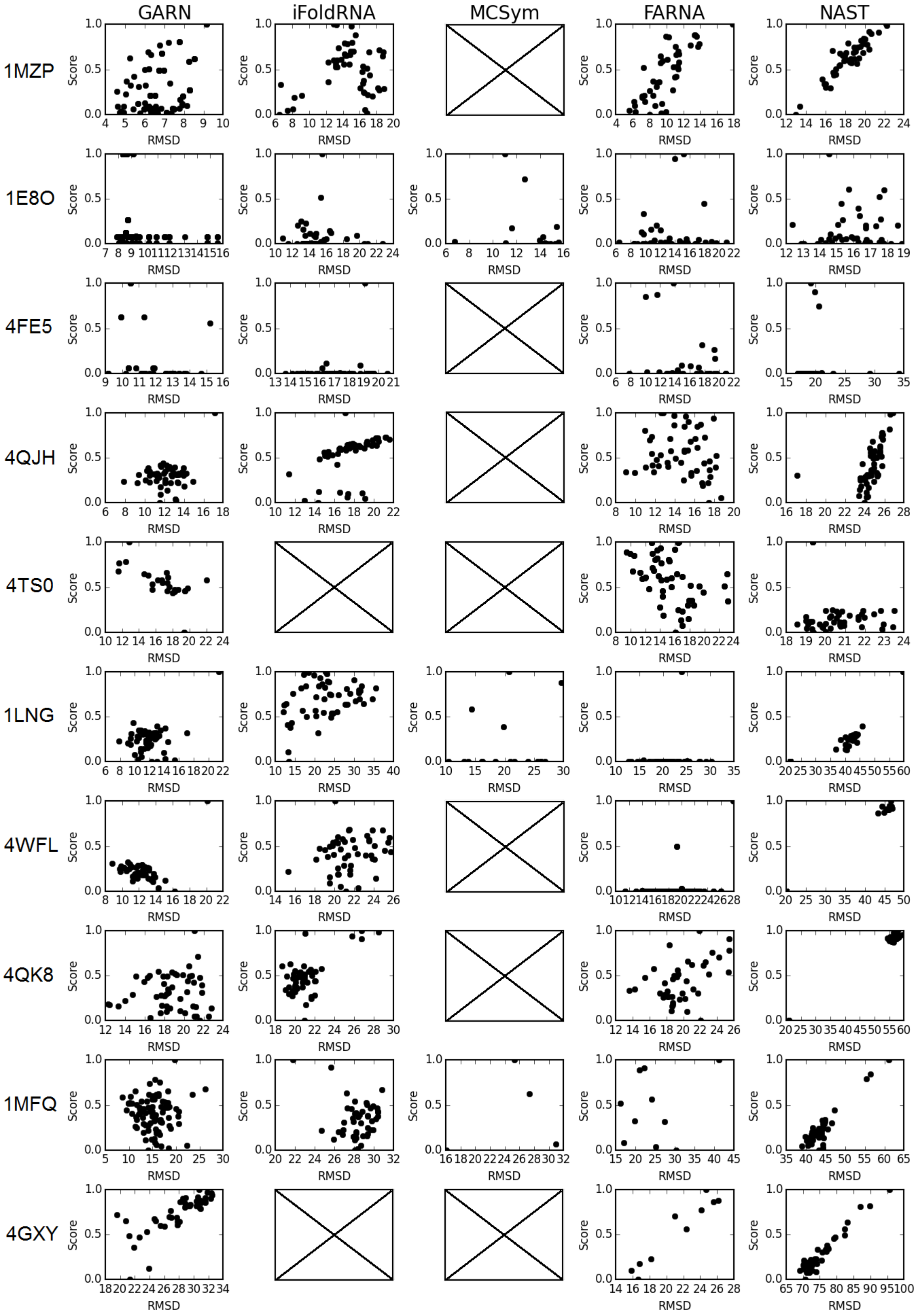

Figure S12: **Energy vs. RMSD curves for the *test set*.** Energy is calculated as the default GARN score multiplied by -1 and normalized.
