# Supplementary material for: GARN: Sampling RNA 3D Structure Space with Game Theory and Knowledge-Based Scoring Strategies
Source: PLoS One. 2015 Aug 27;10(8):e0136444. doi: 10.1371/journal.pone.0136444 (PMC4551674; doi:10.1371/journal.pone.0136444)
Supplement: S13 Fig — Energy vs. RMSD curves for the test set. Each plot shows the default energy scheme for each technique. (PDF) [file pone.0136444.s013.pdf]

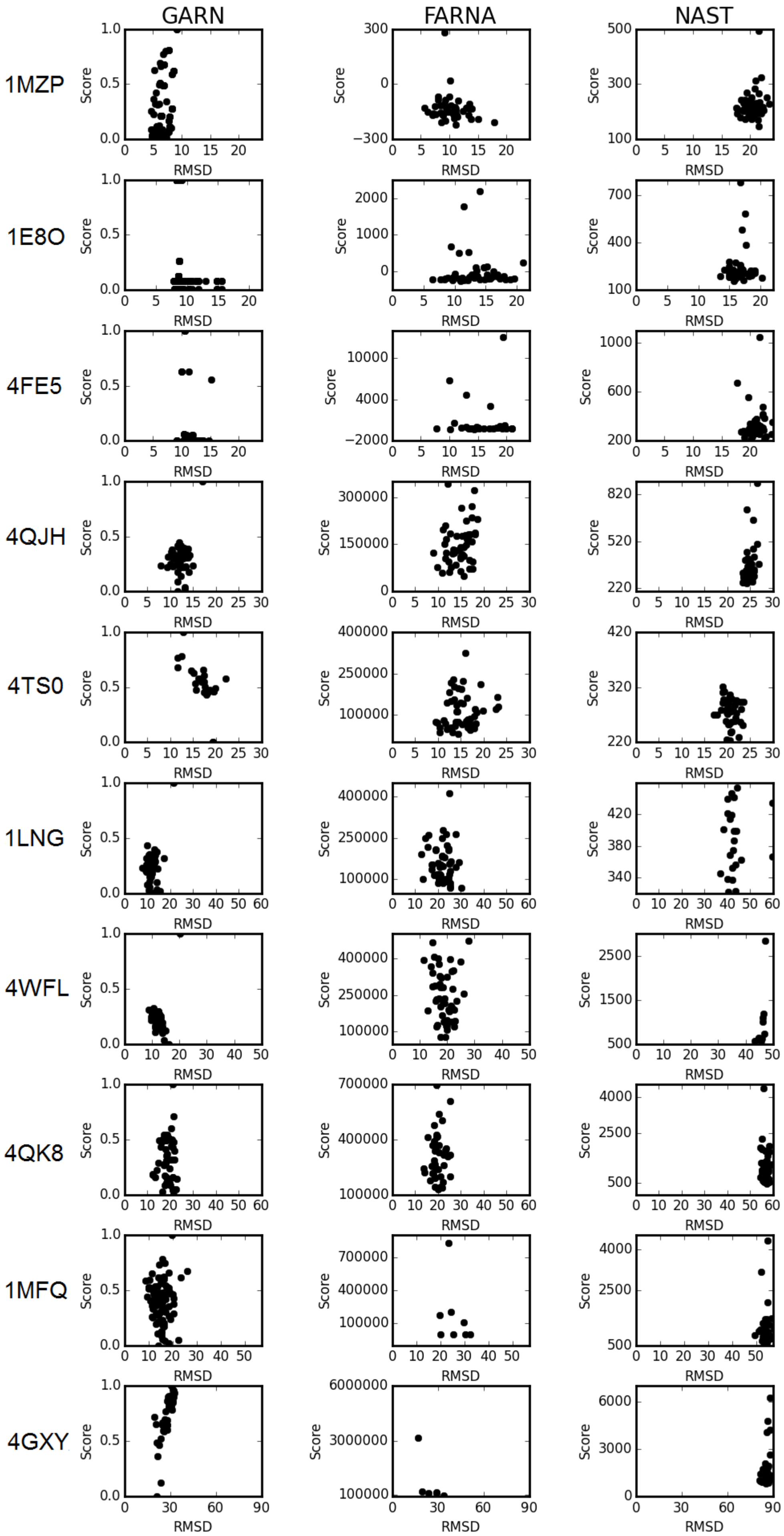

Figure S13: **Energy vs. RMSD curves for the *test set*.** Energy vs. RMSD curves for the *test set*. Each plot shows the default energy scheme for each technique.
