# Supplementary material for: GARN: Sampling RNA 3D Structure Space with Game Theory and Knowledge-Based Scoring Strategies
Source: PLoS One. 2015 Aug 27;10(8):e0136444. doi: 10.1371/journal.pone.0136444 (PMC4551674; doi:10.1371/journal.pone.0136444)
Supplement: S1 Table — The reference set contains 76 molecules of different sizes. (PDF) [file pone.0136444.s014.pdf]

| PDB ID | Description | Biological Family | Excluding reason |
|---|---|---|---|
| 1DQF | 5S rRNA | Ribosomal RNA | |
| 1MSY | Sarcin/Ricin domain from 23s RNA | Ribosomal RNA | |
| 1Q93 | Sarcin/Ricin domain mutant from 28s RNA | Ribosomal RNA | |
| 1T0D | Ribosomal decoding site | Ribosomal RNA | |
| 1T0E | Ribosomal decoding site | Ribosomal RNA | |
| 2OE6 | Ribosomal decoding A site | Ribosomal RNA | |
| 357D | 5S rRNA | Ribosomal RNA | |
| 1SDR | Shine-Dalgarno region of 16S rRNA | Ribosomal RNA | Too small |
| 353D | 5S rRNA | Ribosomal RNA | Too small |
| 361D | 5S rRNA | Ribosomal RNA | Too small |
| 1MME | Hammerhead ribozyme | Ribozymes | |
| 1NUJ | Leadzyme | Ribozymes | |
| 1NBS | Ribonuclease P RNA | Ribozymes | 4-way or more |
| 1U9S | Ribonuclease P RNA | Ribozymes | 4-way or more |
| 1X9C | Hairpin Ribozyme | Ribozymes | 4-way or more |
| 1X9K | Hairpin Ribozyme | Ribozymes | 4-way or more |
| 1Y0Q | Group I ribozyme | Ribozymes | 4-way or more |
| 2A2E | Ribonuclease P RNA | Ribozymes | 4-way or more |
| 2A64 | Ribonuclease P RNA | Ribozymes | 4-way or more |
| 1X8W | Tetrahymena ribozyme | Ribozymes | Unpaired ends |
| 1YKQ | Diels-Alder Ribozyme | Ribozymes | Unpaired ends |
| 2H0S | glmS ribozyme | Ribozymes | Unpaired ends |
| 1D4R | Human SRP helix 6 | SRP RNAs | |
| 1DUH | 4.5s RNA | SRP RNAs | |
| 1FIR | HIV reverse-transcription primer tRNA(Lys,3) | Transfer RNAs | 4-way or more |
| 1I9V | Phenylalanine tRNA | Transfer RNAs | 4-way or more |
| 1YFG | Yeast initiator tRNA | Transfer RNAs | 4-way or more |
| 2TRA | Yeast aspartic acid tRNA | Transfer RNAs | Too small |
| 434D | acceptor stem of tRNA(Ala) from Escherichia coli | Transfer RNAs | Too small |
| 1CSL | HIV-1 rev binding site | Viral RNAs | |
| 1DUQ | HIV-1 rev binding site | Viral RNAs | |
| 1JZV | Bulged RNA from the SL2 stem-loop of the HIV-1 psi-RNA | Viral RNAs | |
| 1XJR | SARS virus genome stem-loop II motif (s2m) | Viral RNAs | |
| 2NOK | IRES domain from hepatitis C virus | Viral RNAs | |
| 397D | HIV-1 trans-activation response region RNA stem | Viral RNAs | |
| 1KH6 | RNA domain essential to HCV IRES-mediated translation initiation | Viral RNAs | 4-way or more |
| 1K9W | HIV-1 RNA dimerization initiation site | Viral RNAs | Too small |
| 1L2X | Viral (beet western yellow virus) rna pseudoknot | Viral RNAs | Too small |
| 2B8R | HIV-1 RNA dimerization initiation site | Viral RNAs | Too small |
| 2IL9 | Ribosomal Binding Domain of the IRES RNA | Viral RNAs | Unpaired ends |
| 157D | RNA duplex | Other RNA | |
| 1F1T | RNA duplex | Other RNA | |
| 1I9X | snRNA | Other RNA | |
| 1J9H | RNA nonamer | Other RNA | |
| 1KD5 | RNA duplex | Other RNA | |
| 1KXK | Ai5gamma group II self-splicing intron | Other RNA | |
| 1QBP | RNA duplex | Other RNA | |
| 1SA9 | RNA octamer | Other RNA | |
| 1Z43 | SRP19 RNA | Other RNA | |
| 205D | RNA duplex | Other RNA | |
| 255D | RNA duplex | Other RNA | |
| 280D | RNA duplex | Other RNA | |
| 333D | RNA octamer | Other RNA | |
| 387D | RNA pseudoknot with 3d domain swapping (inhibit HIVRT) | Other RNA | |
| 402D | RNA octamer | Other RNA | |
| 405D | RNA duplex | Other RNA | |
| 406D | RNA duplex | Other RNA | |
| 409D | RNA octamer | Other RNA | |
| 413D | RNA duplex | Other RNA | |
| 1F27 | RNA pseudoknot | Other RNA | Too small |
| 1G2J | RNA duplex containing phenyl-ribonucleotides | Other RNA | Too small |
| 1KFO | RNA duplex | Other RNA | Too small |
| 1L3Z | RNA heptamer | Other RNA | Too small |

| PDB ID | Description | Biological Family | Excluding reason |
|---|---|---|---|
| 1MHK | 26-nucleotide RNA containing a hook-turn | Other RNA | Too small |
| 1P79 | Bulged RNA tetraplex | Other RNA | Too small |
| 1YZD | RNA duplex | Other RNA | Too small |
| 259D | RNA octamer | Other RNA | Too small |
| 2A0P | RNA octamer | Other RNA | Too small |
| 2AO5 | RNA duplex | Other RNA | Too small |
| 2G32 | RNA octamer | Other RNA | Too small |
| 2G3S | RNA octamer | Other RNA | Too small |
| 2G91 | RNA octamer | Other RNA | Too small |
| 377D | Alternating A-RNA hexamer | Other RNA | Too small |
| 433D | RNA duplex | Other RNA | Too small |
| 438D | RNA nonamer | Other RNA | Too small |
| 472D | Octamer RNA with G.G/U.U tandem wobble base pairs | Other RNA | Too small |

Table ST1: **Reference Set.** The *reference set* contains 76 molecules of different sizes.
