# Supplementary material for: GARN: Sampling RNA 3D Structure Space with Game Theory and Knowledge-Based Scoring Strategies
Source: PLoS One. 2015 Aug 27;10(8):e0136444. doi: 10.1371/journal.pone.0136444 (PMC4551674; doi:10.1371/journal.pone.0136444)
Supplement: S2 Table — Comparison of the RMSD ranges obtained for different methods with the evaluation set. Minimum values of RMSD for each example are shown in blue. (PDF) [file pone.0136444.s015.pdf]

PDB ID	# Nucleotides	# Players	GARN<br>Min	Max	MCSYM<br>Min	Max	Farna<br>Min	Max	NAST<br>Min	Max
1SA9	16	3	3.97	3.97	0.67	3.31	**0.24**	4.22	0.34	2.24
333D	16	3	3.81	3.81	**0.34**	3.57	0.48	3.3	0.36	2.48
402D	16	3	4.15	4.15	**0.14**	4.7	0.29	3.81	NA	NA
409D	16	3	3.93	3.93	0.49	2.95	**0.31**	4.09	0.56	2.30
1JZV	17	3	4.3	9.24	**0.15**	3.94	0.82	6.11	2.51	4.86
1J9H	18	5	4.56	11.53	**1.14**	4.2	1.76	6.77	2.84	4.91
1DQF	19	4	5.39	5.39	**0.84**	3.49	1.46	8.58	NA	NA
1KD5	22	5	9.54	9.54	NA	NA	**1.56**	12.68	1.81	5.71
205D	24	3	4.79	4.79	**0.22**	4.62	0.24	5.1	0.26	2.70
255D	24	3	5.4	5.4	0.66	3.86	**0.21**	6.07	0.32	3.14
280D	24	3	4.36	4.36	**0.22**	5.22	0.28	6.09	**0.22**	2.10
157D	24	5	2.17	12.94	**0.66**	3.31	0.77	7.66	1.11	4.68
1NUJ	24	5	7.07	7.07	NA	NA	**1.62**	10.48	2.07	6.01
1DUQ	26	5	4.86	6.16	**0.66**	7.24	2.41	9.03	NA	NA
1I9X	26	5	6.74	8.35	**1.82**	5.52	3.26	9.25	4.37	9.64
387D	26	5	7.69	7.69	NA	NA	3.95	9.96	**3.63**	7.88
413D	26	5	8.44	8.44	NA	NA	**1.17**	13.29	4.23	6.62
1Q93	27	4	7.66	7.66	NA	NA	0.98	9.64	**0.90**	5.85
1MSY	27	5	4.71	10.06	NA	NA	**1.27**	8.3	1.58	5.54
397D	27	6	4.09	10.16	NA	NA	**2.32**	8.73	3.15	6.04
1QBP	28	5	4.2	8.98	**1.02**	6.35	1.99	8.3	2.07	4.97
1CSL	28	7	3.86	9.69	NA	NA	**2.96**	11.99	5.86	8.84
405D	32	5	2.43	4.73	**0.62**	2.64	1.18	8.5	3.42	6.63
1T0D	33	8	**3.14**	13.87	NA	NA	NA	NA	3.67	7.06
2OE6	33	8	4.77	14.12	NA	NA	NA	NA	**4.67**	7.33
406D	34	5	3.47	9.7	NA	NA	NA	NA	**3.13**	4.87
1T0E	35	9	**3.53**	14.66	NA	NA	NA	NA	3.97	6.49
1F1T	38	7	3.68	9.37	**2.17**	6.86	NA	NA	4.17	7.48
1KFO	38	11	6.39	14.91	NA	NA	NA	NA	**5.99**	10.72
1MME	41	8	**5.76**	10.44	NA	NA	NA	NA	NA	NA
2NOK	44	12	**4.88**	16.77	NA	NA	NA	NA	6.48	10.65
1XJR	47	12	6.23	13.16	5.48	10.82	NA	NA	**5.44**	9.33
1D4R	57	10	8.07	21.61	NA	NA	NA	NA	**4.17**	9.03
357D	60	8	12.17	25.42	5.27	23.23	NA	NA	**3.28**	10.85
1KXK	70	10	8.35	21.28	**5.06**	12.07	NA	NA	6.80	14.00
1DUH	90	13	**8.63**	33.33	NA	NA	NA	NA	NA	NA
1Z43	101	18	**8.98**	15.54	NA	NA	NA	NA	31.24	55.70

Table ST2: **Comparison of RMSD values.** Comparison of the RMSD ranges obtained for different methods with the *evaluation set*. Minimum values of RMSD for each example are shown in blue.
