# Supplementary material for: GARN: Sampling RNA 3D Structure Space with Game Theory and Knowledge-Based Scoring Strategies
Source: PLoS One. 2015 Aug 27;10(8):e0136444. doi: 10.1371/journal.pone.0136444 (PMC4551674; doi:10.1371/journal.pone.0136444)
Supplement: S3 Table — The test set contains 10 molecules of different sizes. (PDF) [file pone.0136444.s016.pdf]

PDB ID	Description	# Nucleotides	# of 3- and 4-way junctions
1E8O	7SL RNA	49	One 3-way
1MZP	Fragment of 23S rRNA	55	None
4FE5	Guanine riboswitch aptamer domain	67	One 3-way
4QJH	Twister Ribozyme	74	One 3-way
4TS0	Spinach RNA aptamer	89	None
1LNG	7S.S SRP RNA	97	One 3-way
4WFL	Bacterial SRP Alu domain	107	Two 3-way
4QK8	C-di-AMP riboswitch	124	Two 3-way
1MFQ	7S RNA of human SRP	127	One 3-way
4GXY	Adenosylcobalamin riboswitch	172	One 4-way and one 3-way

Table ST3: **Test set.** The *test set* contains 10 molecules of different sizes.
