# Supplementary material for: GARN: Sampling RNA 3D Structure Space with Game Theory and Knowledge-Based Scoring Strategies
Source: PLoS One. 2015 Aug 27;10(8):e0136444. doi: 10.1371/journal.pone.0136444 (PMC4551674; doi:10.1371/journal.pone.0136444)
Supplement: S4 Table — The molecule contains 49 nucleotides and 8 players. Six different parameter sets per game type are shown. Values shown in blue highlight combinations providing conformations with RMSD values below 8Å. Elements highlighted in yellow correspond to the default GARN options for the molecule. (PDF) [file pone.0136444.s017.pdf]

Score	Frozen	Algorithm	Game	# conformations w/ RMSD <10 Å	RMSD range
**Lennard-Jones**	**No**	**EXP3**	**AA**	**2**	**6.82-15.40**
			OA	0	12.1-15.65
			OO	3	7.96-12
		UCB	AA	1	8.1-12.8
			OA	1	8.91-11.71
			OO	0	11.9-12.42
	Yes	EXP3	AA	3	8.75-12.36
			OA	3	7.7-12.4
			OO	3	8.89-11.9
		UCB	AA	2	8.41-12.02
			OA	1	8.67-12.69
			OO	2	8.89-12.92
Modified Lennard-Jones	No	EXP3	AA	3	8.75-12
			OA	3	8.26-11.6
			OO	3	8.01-11.1
		UCB	AA	2	8.26-15.21
			OA	1	8.14-14.9
			OO	0	11.9-11.9
	Yes	EXP3	AA	3	8.75-12.36
			OA	4	7.71-9.33
			OO	4	8.75-9.33
		UCB	AA	2	8.26-15.53
			OA	1	8.64-16.2
			OO	0	11.65-12.92
Gauss	No	EXP3	AA	4	8.19-9.34
			OA	3	8.41-12.92
			OO	2	8.19-15.2
		UCB	AA	2	8.41-11.88
			OA	2	8.37-10.88
			OO	0	11.9-11.9
	Yes	EXP3	AA	4	8.75-9.34
			OA	1	8.9-11.88
			OO	1	8.75-12.92
		UCB	AA	1	8.41-15.53
			OA	4	8.41-8.93
			OO	2	8.31-12.92
$1/d^2$	No	EXP3	AA	3	8.51-10.64
			OA	2	7.38-14.9
			OO	2	8.89-12.3
		UCB	AA	4	8.08-9.81
			OA	4	6.66-8.72
			OO	2	8.81-11.9
	Yes	EXP3	AA	4	8.19-9.33
			OA	1	8.64-11.6
			OO	2	8.26-16.2
		UCB	AA	3	7.44-10
			OA	3	8.64-14.92
			OO	1	9.75-12.92

Table ST4: **Sampling results for the structure of the core of the ALU domain of the mammalian SRP (PDB ID 1E8O).** The molecule contains 49 nucleotides and 8 players. Six different parameter sets per game type are shown. Values shown in blue highlight combinations providing conformations with RMSD values below 8Å. Elements highlighted in yellow correspond to the default GARN options for the molecule.
