# Supplementary material for: GARN: Sampling RNA 3D Structure Space with Game Theory and Knowledge-Based Scoring Strategies
Source: PLoS One. 2015 Aug 27;10(8):e0136444. doi: 10.1371/journal.pone.0136444 (PMC4551674; doi:10.1371/journal.pone.0136444)
Supplement: S5 Table — The molecule contains 55 nucleotides and 8 players. Six different parameter sets per game type are shown. Values shown in blue highlight combinations providing conformations with RMSD values below 5Å. Elements highlighted in yellow correspond to the default GARN options for the molecule. (PDF) [file pone.0136444.s018.pdf]

Score	Frozen	Algorithm	Game	# conformations w/ RMSD <7 Å	RMSD range
Lennard-Jones	No	EXP3	AA	1	6.84-11.62
			OA	6	4.61-11.67
			OO	7	4.71-11.23
		UCB	AA	5	5.24-11.23
			OA	10	4.46-9.31
			OO	12	7.64-16.6
	Yes	EXP3	AA	2	6.84-11.17
			OA	11	4.91-8.07
			OO	9	4.22-9.58
		UCB	AA	10	4.32-11.37
			OA	8	4.27-10.12
			OO	10	6.41-15.68
**Modified Lennard-Jones**	No	EXP3	AA	3	5.43-11.85
			OA	7	5.25-8.72
			OO	13	4.46-8.68
		UCB	AA	12	4.32-8.86
			OA	12	4.4-9.38
			OO	6	4.27-11.23
	**Yes**	EXP3	AA	4	5.92-10.07
			OA	13	4.43-8.5
			OO	10	5.25-9.72
		**UCB**	AA	17	4.3-7.26
			**OA**	**11**	**4.32-10.58**
			OO	6	4.32-11.85
Gauss	No	EXP3	AA	6	5.21-10.07
			OA	12	5.72-8.98
			OO	8	5.34-9.72
		UCB	AA	8	4.3-11.85
			OA	5	5.68-11.72
			OO	10	4.88-9.58
	Yes	EXP3	AA	6	8.49-16.92
			OA	10	4.61-9.47
			OO	9	4.46-10.07
		UCB	AA	4	4.95-10.62
			OA	5	4.81-11.08
			OO	15	4.3-9.72
$1/d^2$	No	EXP3	AA	6	5.69-10.07
			OA	1	5.77-11.85
			OO	10	4.27-8.3
		UCB	AA	2	5.84-10.07
			OA	2	6.34-11.67
			OO	19	3.63-9.14
	Yes	EXP3	AA	6	5.3-10.07
			OA	2	5.77-11.08
			OO	8	5.24-8.64
		UCB	AA	2	4.4-11.85
			OA	2	4.79-10.52
			OO	13	3.46-10.5

Table ST5: **Sampling results for the structure of the L1 protuberance in the ribosome (PDB ID 1MZP).** The molecule contains 55 nucleotides and 8 players. Six different parameter sets per game type are shown. Values shown in blue highlight combinations providing conformations with RMSD values below 5Å. Elements highlighted in yellow correspond to the default GARN options for the molecule.
