# Supplementary material for: GARN: Sampling RNA 3D Structure Space with Game Theory and Knowledge-Based Scoring Strategies
Source: PLoS One. 2015 Aug 27;10(8):e0136444. doi: 10.1371/journal.pone.0136444 (PMC4551674; doi:10.1371/journal.pone.0136444)
Supplement: S6 Table — The molecule contains 67 nucleotides and 13 players. Six different parameter sets per game type are shown. Values shown in blue highlight combinations providing conformations with RMSD values below 8Å. Elements highlighted in yellow correspond to the default GARN options for the molecule. (PDF) [file pone.0136444.s019.pdf]

Score	Frozen	Algorithm	Game	# conformations w/ RMSD <11 Å	RMSD range
Lennard-Jones	No	EXP3	AA	8	**7.14-14.53**
			OA	8	6.8-20.31
			OO	8	8.23-19.1
		UCB	AA	6	6.42-15.88
			OA	4	8.98-19.17
			OO	12	7.64-16.6
	Yes	EXP3	AA	5	9.03-14.52
			OA	2	9.45-16.77
			OO	3	6.79-18.94
		UCB	AA	6	6.42-15.88
			OA	4	7.85-18.36
			OO	10	6.41-15.68
Modified Lennard-Jones	No	EXP3	AA	8	8.21-13.73
			OA	5	8.09-17.66
			OO	2	9.38-18.48
		UCB	AA	1	9.15-17.21
			OA	7	8.76-17.04
			OO	4	7.22-17.32
	Yes	EXP3	AA	7	8.76-14.7
			OA	9	8.45-18.88
			OO	4	7.98-20.46
		UCB	AA	8	8.27-17.7
			OA	9	8.11-18.27
			OO	4	9.59-20.12
Gauss	No	EXP3	AA	1	10.91-17.95
			OA	9	8.45-18.88
			OO	3	9.47-21.18
		UCB	AA	6	6.67-15.69
			OA	7	6.54-16.52
			OO	3	10.09-18.02
	Yes	EXP3	AA	6	8.49-16.92
			OA	5	9-20.69
			OO	5	7.85-21.82
		UCB	AA	6	9-17.29
			OA	2	9.92-20.69
			OO	5	8.43-19.21
$1/d^2$	No	EXP3	AA	0	11.19-16.93
			OA	8	7.67-18.82
			OO	1	9.11-20.1
		UCB	AA	6	8.65-16.48
			OA	5	8.6-16.68
			OO	6	8.86-18.46
	Yes	EXP3	AA	3	7.25-16.92
			OA	12	7.55-17.74
			OO	4	8.23-20.63
		UCB	AA	8	8.28-16.18
			OA	9	8.89-18.47
			OO	5	7.2-19.09

Table ST6: **Sampling results for the xpt-pbuX guanine riboswitch aptamer domain (PDB ID 4FE5).** The molecule contains 67 nucleotides and 13 players. Six different parameter sets per game type are shown. Values shown in blue highlight combinations providing conformations with RMSD values below 8Å. Elements highlighted in yellow correspond to the default GARN options for the molecule.
