# Supplementary material for: GARN: Sampling RNA 3D Structure Space with Game Theory and Knowledge-Based Scoring Strategies
Source: PLoS One. 2015 Aug 27;10(8):e0136444. doi: 10.1371/journal.pone.0136444 (PMC4551674; doi:10.1371/journal.pone.0136444)
Supplement: S7 Table — The molecule contains 127 nucleotides and 24 players. Six different parameter sets per game type are shown. Values shown in blue highlight combinations providing conformations with RMSD values below 11Å. Elements highlighted in yellow correspond to the default GARN options for the molecule. (PDF) [file pone.0136444.s020.pdf]

Score	Frozen	Algorithm	Game	# conformations w/ RMSD <13 Å	RMSD range
**Lennard-Jones**	**No**	**EXP3**	**AA**	**8**	**9.68-20.64**
			OA	0	17.59-27.65
			OO	0	14.8-32.25
		UCB	AA	0	14.65-26.16
			OA	1	12.71-27.07
			OO	2	11.99-29.58
	Yes	EXP3	AA	3	11.51-22.35
			OA	0	18.17-25.05
			OO	0	14.29-27.13
		UCB	AA	3	10.18-31.56
			OA	0	15.18-28.79
			OO	0	14.08-29.4
**Modified Lennard-Jones**	**No**	**EXP3**	**AA**	**5**	**10.04-25.07**
			OA	0	16.04-26.36
			OO	0	16.19-24.80
		UCB	AA	0	13.93-27.64
			OA	0	17.08-28.31
			OO	0	14.08-29.4
	Yes	EXP3	AA	0	13.03-20.26
			OA	1	11.79-28.80
			OO	0	18.76-31.88
		UCB	AA	0	13.55-29.04
			OA	0	14.89-25.59
			OO	0	14.94-28.14
Gauss	No	EXP3	AA	6	10.07-29.88
			OA	0	15.60-27.91
			OO	1	12.76-26.78
		UCB	AA	0	15.59-29.68
			OA	0	17.51-27.98
			OO	0	16.69-29.6
	Yes	EXP3	AA	4	10.08-27.64
			OA	0	15.82-23.24
			OO	0	22.7-24.61
		UCB	AA	0	16.75-27.61
			OA	0	17.12-29.65
			OO	2	10.77-28.16
$1/d^2$	No	EXP3	AA	8	10.74-22.13
			OA	1	11.71-23.85
			OO	0	16.13-21.79
		UCB	AA	0	18.86-26.35
			OA	0	17.91-23.79
			OO	0	16.24-26.99
	Yes	EXP3	AA	3	9.83-23.79
			OA	0	15.52-27.85
			OO	0	18.73-34.37
		UCB	AA	0	16.24-26.12
			OA	0	17.25-26.38
			OO	0	16.74-27.35

Table ST7: **Sampling results for the structure of the core of the ternary S-domain complex of human signal recognition particle (PDB ID 1MFQ).** The molecule contains 127 nucleotides and 24 players. Six different parameter sets per game type are shown. Values shown in blue highlight combinations providing conformations with RMSD values below 11Å. Elements highlighted in yellow correspond to the default GARN options for the molecule.
