# Supplementary material for: GARN: Sampling RNA 3D Structure Space with Game Theory and Knowledge-Based Scoring Strategies
Source: PLoS One. 2015 Aug 27;10(8):e0136444. doi: 10.1371/journal.pone.0136444 (PMC4551674; doi:10.1371/journal.pone.0136444)
Supplement: S8 Table — The molecule contains 172 nucleotides and 32 players. Four different parameter sets per game type are shown. As the molecule is relatively large, due to computing time constraints, extensive results are reported only for the AA game. Values shown in blue highlight combinations providing conformations with RMSD values below 15Å. Elements highlighted in yellow correspond to the default GARN options for the molecule. (PDF) [file pone.0136444.s021.pdf]

Score	Frozen	Algorithm	Game	# conformations w/ RMSD <18 Å	RMSD range
Lennard-Jones	No	EXP3	AA	0	24.32-36.17
		UCB	AA	2	17.44-27.77
	Yes	EXP3	AA	0	19.43-35.12
		UCB	AA	1	17.79-30.89
**Modified Lennard-Jones**	**No**	**EXP3**	**AA**	**3**	**14.27-31.04**
		UCB	AA	0	18.75-24.68
	Yes	EXP3	AA	7	14.61-24.58
		UCB	AA	0	18.27-26.45
Gauss	No	EXP3	AA	4	13.31-30.9
		UCB	AA	3	15.49-26.88
	Yes	EXP3	AA	4	15.8-31.18
		UCB	AA	1	17.49-27.54
$1/d^2$	No	EXP3	AA	3	16.51-23.99
		UCB	AA	1	17.02-26.3
	Yes	EXP3	AA	9	14.99-28.35
		UCB	AA	0	21.70-26.91

Table ST8: **Sampling results for the structure of the adenosylcobalamin riboswitch (PDB ID 4GXY).** The molecule contains 172 nucleotides and 32 players. Four different parameter sets per game type are shown. As the molecule is relatively large, due to computing time constraints, extensive results are reported only for the AA game. Values shown in blue highlight combinations providing conformations with RMSD values below 15Å. Elements highlighted in yellow correspond to the default GARN options for the molecule.
