# Supplementary material for: GARN: Sampling RNA 3D Structure Space with Game Theory and Knowledge-Based Scoring Strategies
Source: PLoS One. 2015 Aug 27;10(8):e0136444. doi: 10.1371/journal.pone.0136444 (PMC4551674; doi:10.1371/journal.pone.0136444)
Supplement: S9 Table — The default gameplay scheme is based on the existence of high-order junctions, and on the ratio of the number of players in helices to the number of players in junctions. (PDF) [file pone.0136444.s022.pdf]

PDB ID	# Nucleotides	# Players	Helix/Junction Ratio	# 3-way or 4-way junctions	Default Gameplay (Game/Algorithm/Score)
1MZP	55	8	1.3	0	OA/UCB/Modified Lennard-Jones
4TS0	89	21	1.28	0	OA/UCB/Modified Lennard-Jones
1E8O	49	8	2	1 (3-way)	AA/EXP3/Lennard-Jones
1LNG	97	16	1.6	1 (3-way)	AA/EXP3/Lennard-Jones
4WFL	107	18	1.8	2 (3-way)	AA/EXP3/Lennard-Jones
4FE5	67	14	1.75	1 (3-way)	AA/EXP3/Lennard-Jones
4QJH	72	15	1.4	1 (3-way)	AA/EXP3/Modified Lennard-Jones
4QK8	124	20	1.3	2 (3-way)	AA/EXP3/Modified Lennard-Jones
1MFQ	127	24	1.44	1 (3-way)	AA/EXP3/Modified Lennard-Jones
4GXY	172	32	1.15	1 (3-way) and 1 (4-way)	AA/EXP3/Modified Lennard-Jones.

Table ST9: **Gameplay settings for the test set.** The default gameplay scheme is based on the existence of high-order junctions, and on the ratio of the number of players in helices to the number of players in junctions.
