# Supplementary material for: GARN: Sampling RNA 3D Structure Space with Game Theory and Knowledge-Based Scoring Strategies
Source: PLoS One. 2015 Aug 27;10(8):e0136444. doi: 10.1371/journal.pone.0136444 (PMC4551674; doi:10.1371/journal.pone.0136444)
Supplement: S10 Table — Elements in blue indicate the results for the GARN default settings. (PDF) [file pone.0136444.s023.pdf]

PDB ID	# Nucleotides	# Players	H/J Ratio	RMSD	GARN AA/EXP3/LJ	GARN AA/EXP3/MLJ	GARN OA/UCB/MLJ	iFoldRNA	MCSym	Farna	NAST
1MZP	55	8	1.3	min	6.84	5.43	4.32	8.23	NA	5.61	12.97
				max	11.62	11.85	10.58	12.68	NA	17.80	26.38
				# of samples with RMSD < 5Å (on 50 samples)	1	3	7	0	–	0	0
4TS0	89	21	1.28	min	11.98	10.53	10.42	NA	NA	9.47	19.84
				max	22.50	18.31	23.13	NA	NA	23.19	22.79
				# of samples with RMSD < 5Å (on 50 samples)	1	2	4	–	–	4	0
1E8O	49	8	2	min	6.82	8.75	8.64	12.62	6.75	7.78	12.34
				max	15.40	12	16.2	17.71	15.55	21.02	20.29
				# of samples with RMSD < 8Å (on 50 samples)	13	0	0	0	1	1	0
4FE5	67	14	1.75	min	7.14	8.21	8.11	15.04	NA	7.80	17.00
				max	14.53	13.73	18.27	20.12	NA	20.98	34.17
				# of samples with RMSD < 9Å (on 50 samples)	2	2	3	0	–	1	0
4QJH	72	15	1.4	min	7.87	10.56	10.13	11.34	NA	8.99	23.44
				max	14.93	15.31	20.32	21.24	NA	18.67	26.57
				# of samples with RMSD < 9Å (on 50 samples)	3	0	0	0	–	1	0
1LNG	97	16	1.6	min	9.11	7.85	10.13	11.92	10.52	12.67	36.49
				max	17.63	17.07	20.32	35.53	29.58	30.19	59.66
				# of samples with RMSD < 10Å (on 50 samples)	1	6	0	0	–	0	0
4WFL	107	18	1.8	min	9.79	8.82	14.70	18.08	NA	11.33	43.41
				max	17.21	16.22	21.38	25.75	NA	26.00	47.04
				# of samples with RMSD < 10Å (on 50 samples)	1	5	0	0	–	0	0
4QK8	124	20	1.3	min	12.25	13.70	13.23	18.66	NA	13.61	54.43
				max	22.78	23.13	22.19	28.49	NA	29.41	59.44
				# of samples with RMSD < 14Å (on 50 samples)	3	1	1	0	–	1	0
1MFQ	127	24	1.44	min	10.49	9.68	14.89	20.42	16.07	16.13	38.91
				max	21.05	20.64	25.59	34.08	30.97	41.27	44.17
				# of samples with RMSD < 11Å (on 50 samples)	3	7	0	0	–	0	0
4GXY	172	32	1.15	min	24.32	14.27	21.99	NA	NA	15.84	69.04
				max	36.17	31.04	38.13	NA	NA	26.17	74.83
				# of samples with RMSD < 15Å (on 50 samples)	0	2	NA	0	–	0	0

Table ST10: **Comparison with other methods.** Elements in blue indicate the results for the GARN default settings.
