# Supplementary material for: GARN: Sampling RNA 3D Structure Space with Game Theory and Knowledge-Based Scoring Strategies
Source: PLoS One. 2015 Aug 27;10(8):e0136444. doi: 10.1371/journal.pone.0136444 (PMC4551674; doi:10.1371/journal.pone.0136444)
Supplement: S11 Table — * indicates that the computation was performed locally on an Intel Xeon E5607 2.27GHz CPU. NAST computation was also performed with OpenCL on a NVIDIA Quadro 5000 GPU. Other computations were performed on dedicated servers. NAST appears to be much faster than the other methods, but it calculates only secondary structure interactions: the lack on tertiary information as input only allows for extended structures as a result. The computation time for RNAJAG was not available from [30]. (PDF) [file pone.0136444.s024.pdf]

PDB ID	# Nucleotides	GARN*		iFoldRNA		MC-SYM		FARNA*		NAST*	
		Time	# structures	Time	# structures	Time	# structures	Time	# structures	Time	# structures
1MZP	55	∼ 5 min	50	∼ 1 h 40	50	NA	NA	∼ 5 min	50	∼ 15 min	50
1E8O	49	∼ 5 min	50	∼ 1 h 30	50	∼ 10 h	18	∼ 5 min	50	∼ 10 min	50
4FE5	67	∼ 30 min	50	∼ 2h30	50	NA	NA	∼ 4 h	43	∼ 20 min	50
4QJH	74	∼ 30 min	50	∼ 2h	50	NA	NA	∼ 4 h 30	50	∼ 20 min	50
4TS0	89	∼ 1h	50	∼ NA	NA	∼ 8 h	50	∼ 4 h	50	∼ 25 min	50
1LNG	97	∼ 1h	50	∼ 3h	50	∼ 8 h	50	∼ 4 h	50	∼ 25 min	50
4WFL	107	∼ 2h	50	∼ 3h	50	NA	NA	∼4 h 30	50	∼ 25 min	50
4QK8	124	∼ 2h	50	∼ 4h	50	NA	NA	∼ 5 h	50	∼ 25 min	50
1MFQ	127	∼ 2 h	50	∼ 4 h 30	50	∼ 6 h	4	∼ 5 h	8	∼ 25 min	50
4GXY	172	∼ 12 h	50	NA	NA	NA	NA	∼ 30 h	6	∼ 25 min	50

Table ST11: **Computation time and number of output pdb files generated for all the compared methods.** * indicates that the computation was performed locally on an Intel Xeon E5607 2.27GHz CPU. NAST computation was also performed with OpenCL on a NVIDIA Quadro 5000 GPU. Other computations were performed on dedicated servers. NAST appears to be much faster than the other methods, but it calculates only secondary structure interactions: the lack on tertiary information as input only allows for extended structures as a result. The computation time for RNAJAG was not available from [30].
