# Supplementary material for: GARN: Sampling RNA 3D Structure Space with Game Theory and Knowledge-Based Scoring Strategies
Source: PLoS One. 2015 Aug 27;10(8):e0136444. doi: 10.1371/journal.pone.0136444 (PMC4551674; doi:10.1371/journal.pone.0136444)
Supplement: S12 Table — GARN RMSD values are calculated only for the coarse-grained representation of the three-way junction. RNAJAG RMSD values were obtained from [30]. Elements in blue had the lowest RMSD values. (PDF) [file pone.0136444.s025.pdf]

PDB	Nts	Nodes	GARN min	GARN max	RNAJAG
2FK6	52	11	9.56	11.80	4.01
1DK1	57	13	4.58	6.71	6.16
1MMS	58	11	4.21	6.96	4.13
3EGZ	65	10	6.57	8.13	6.59
3D2G	77	14	4.67	7.52	2.07
2HOJ	78	15	1.99	11.99	2.18
2GDI	80	15	4.58	7.94	1.98
1LNG	97	16	4.99	7.19	9.04
1MFQ	127	24	4.70	5.71	5.26

Table ST12: **Comparison with RNAJAG for three-way junctions.** GARN RMSD values are calculated only for the coarse-grained representation of the three-way junction. RNAJAG RMSD values were obtained from [30]. Elements in blue had the lowest RMSD values.
